# Mucin gel assembly is controlled by a collective action of non-mucin proteins, disulfide bridges, Ca^2**+**^-mediated links, and hydrogen bonding

**DOI:** 10.1038/s41598-018-24223-3

**Published:** 2018-04-11

**Authors:** Oliver W. Meldrum, Gleb E. Yakubov, Mauricio R. Bonilla, Omkar Deshmukh, Michael A. McGuckin, Michael J. Gidley

**Affiliations:** 1 ARC Centre of Excellence in Plant Cell Walls, The University of Queensland, St Lucia, 4072 Qld Australia; 2 Centre for Nutrition and Food Sciences, Queensland Alliance for Agriculture and Food Innovation, The University of Queensland, St Lucia, 4072 Qld Australia; 3 School of Chemical Engineering, The University of Queensland, St Lucia, 4072 Qld Australia; 4 Chronic Disease Biology and Care Program, Mater Research Institute - The University of Queensland, Translational Research Institute, Woolloongabba, QLD 4102 Australia

## Abstract

Mucus is characterized by multiple levels of assembly at different length scales which result in a unique set of rheological (flow) and mechanical properties. These physical properties determine its biological function as a highly selective barrier for transport of water and nutrients, while blocking penetration of pathogens and foreign particles. Altered integrity of the mucus layer in the small intestine has been associated with a number of gastrointestinal tract pathologies such as Crohn’s disease and cystic fibrosis. In this work, we uncover an intricate hierarchy of intestinal mucin (Muc2) assembly and show how complex rheological properties emerge from synergistic interactions between mucin glycoproteins, non-mucin proteins, and Ca^2+^. Using a novel method of mucus purification, we demonstrate the mechanism of assembly of Muc2 oligomers into viscoelastic microscale domains formed via hydrogen bonding and Ca^2+^-mediated links, which require the joint presence of Ca^2+^ ions and non-mucin proteins. These microscale domains aggregate to form a heterogeneous yield stress gel-like fluid, the macroscopic rheological properties of which are virtually identical to that of native intestinal mucus. Through proteomic analysis, we short-list potential protein candidates implicated in mucin assembly, thus paving the way for identifying the molecules responsible for the physiologically critical biophysical properties of mucus.

## Introduction

Mucus is a complex viscoelastic fluid produced by specialized secretory cells in the linings of the respiratory, gastrointestinal and urogenital tracts. Mucus functions as a barrier against infection and dehydration, and as a lubricant, are underpinned by a unique set of physicochemical and mechanical properties, where fluid-like (viscous) and solid-like (elastic) behaviors are required to be in balance to ensure normal physiological function. For example, reduction in viscoelasticity facilitates bacterial migration in the gastric mucus, which is a key mechanism in the development of *Helicobacter pylori* infection^1^. In comparison, hyper-viscous mucus is a central pathogenic feature of cystic fibrosis, where mucus accumulation and stagnation results in airway and intestinal obstruction and chronic bacterial colonization^2,3^. Although it is established the mucus network is formed via supramolecular assembly, the details of interactions remain poorly understood, which limits our ability to translate physical insights into tangible therapeutic interventions^4^. The current model represents mucus assembly as a semi-transient associative polymer network formed by interconnected longer-living cross-links (hydrogen and disulfide bonds) and relatively shorter-living physical entanglements^5–7^.

Despite marked differences in the composition and biophysical properties of mucus at different mucosal surfaces (e.g. lungs versus gastrointestinal tract^8^), there are fundamental similarities in the way gel-forming secretory mucins assemble. The assembly mechanism stems from a common blueprint of their domain structure, which comprises a largely unstructured glycosylated (>50%) central domain flanked by the amino (N-) and carboxyl (C-) termini. The glycosylated region contains a highly heterogeneous array of O-linked oligosaccharides that confer mucin its extended conformation^9,10^. O-linked glycans contain sialic acid (pKa ∼ 2.6) and sulfate (pKa <1) sugar residues so that mucins take on many generic properties of anionic polyelectrolytes, such as sensitivity to pH and ionic strength^11^. C- and N-termini feature von Willebrand assemblies and cysteine-rich globular domains, which are responsible for mucin polymerization through hydrogen bond interactions and the formation of disulfide bridges^12^. The predominant mucin expressed in the small intestine of humans, pigs, rats etc. is MUC2/Muc2. The sequence of human MUC2 corresponds to an apomucin (protein only part) containing ca. 5000 amino acids^13^ that features an extensively glycosylated central domain. The central domain includes a tandem repeat region that due to genetic polymorphism comprises anywhere between 51 to 115 repeat fragments (23 amino acids each)^13^. This polymorphism together with the broad distribution of glycosylation patterns confer MUC2 a wide distribution of molecular weights, with values reported in the literature varying from ∼2.7 MDa^14^ up to ∼7 MDa^15^.

Prior to secretion, mucin is packaged into secretory granules in a highly condensed, water-depleted state through the action of Ca^2+^ ions and cytosol electrolytes that facilitate polymer folding by screening charges on the O-linked glycans^16^. Upon secretion, the effect of electrolyte dilution, such as decrease in Ca^2+^ concentration, drives the swelling of mucin that when unfolded may occupy as much as 100 to 1000-times its initial volume^17^. This secretion-swelling process can hardly, if at all, be reversed; providing one of the reasons why purified mucins do not replicate the viscoelastic and/or gel-like properties of native mucus, as has been shown for salivary, respiratory, and gastrointestinal mucins^18–20^. In addition, the chaotropic and reducing agents used in protein purification may result in partial denaturation of mucin, potentially compromising formation of the associative network.

Finally, we should recognize our inability to adequately describe complex rheological (flow) properties of mucus in simplified terms such as viscosity or viscoelasticity, omitting other aspects of complex supramolecular dynamics such as time-dependent properties (e.g. creep), hysteresis behaviors, spatial heterogeneity, and the dependence of rheological properties on the length scale over which these properties are probed. At the macroscale, mucus behaves as a viscoelastic gel (G′ > G″), yet is distinguished from a classical gel by its response to small and large mechanical deformation as probed by small amplitude oscillatory and steady shear rheological tests, respectively^21^. At the microscale, mucus behaves as a solution of entangled polymers that may or may not display significant elasticity (G′ ≈ G″) depending on the mucin genetic type and glycosylation pattern^22^.

In the current work, we study the complex rheological properties of preparations of Muc2 mucin from the porcine small intestine. Muc2 is the major gel-forming mucin in the intestinal tract mucus layer that provides the structural framework responsible for its function as a physical and diffusion barrier overlaying the mucosa^23^. We probe the molecular and meso-scale interactions of native (contains non-mucin components) and extensively purified Muc2 preparations by investigating their viscoelastic response across a broad range of frequencies using narrow gap rotational rheometry and passive particle tracking microrheology (PTM). Furthermore, we investigate the effect of divalent ions (Ca^2+^), chelating (EDTA), chaotropic (GuHCl), and reducing (DTT) agents, as well as pH on the viscoelastic properties of both native and extensively purified mucins to probe the influence of different components and interactions on the structure and flow properties of mucus. Our findings point towards a key role of non-mucin proteins in the formation of an associative network. We also show how hydrogen bonding, Ca^2+^-mediated cross-links, disulfide bridges and physical entanglements act as a collective to form a hierarchical mucus network responsible for its complex rheological behavior.

## Results

### Characterization of mucin preparations and protein identification

The mucin preparation isolated under non-denaturing conditions (Supplementary Figures S1, S2, and S3) was designated as ND-mucin. The purification procedure avoided the immediate freezing of crude mucus after collection, enabling a substantial increase in the amount of recovered material. The procedure yielded mucin with enhanced viscoelastic behavior, as can be visualized by stretching a small droplet of liquid and observing formation of filaments (‘spinnbarkeit’ test)^24^. In addition, an extensively purified mucin was isolated from mucus using GuHCl according to a well-established procedure and designated as GH-mucin^25^.

Tandem mass spectroscopy of lyophilized products detected Muc2 mucin as the dominant glycoprotein in both ND- and GH-mucin preparations. Muc2 is the only mucin type identified with peptides limited to the non-glycosylated terminal regions (Supplementary Figure S4). ND- and GH-mucin preparations are profiled using peptide mass fingerprinting against the SwissProt database and identified proteins were ranked using ProteinPilot Software on the basis of their relative peptide abundance and presented in Supplementary Tables S1 and S2. The identified proteins represent a complex mixture of digestive enzymes, mucus and dietary proteins, with the remainder representing intracellular/blood plasma proteins. Major blood group and cytosol proteins include albumin, keratin and hemoglobin, which may originate from contamination during the dissection procedure. The cytosol proteins are expected to be present due to the continuous turnover of the epithelium and the consequent release of the cytosol materials into the lumen, which have also been found elsewhere^26^. We note that non-mucin proteins are chemically or physically associated with mucin and thus were retained in the preparation after two rounds of isopycnic density gradient centrifugation.

**Figure 1:**
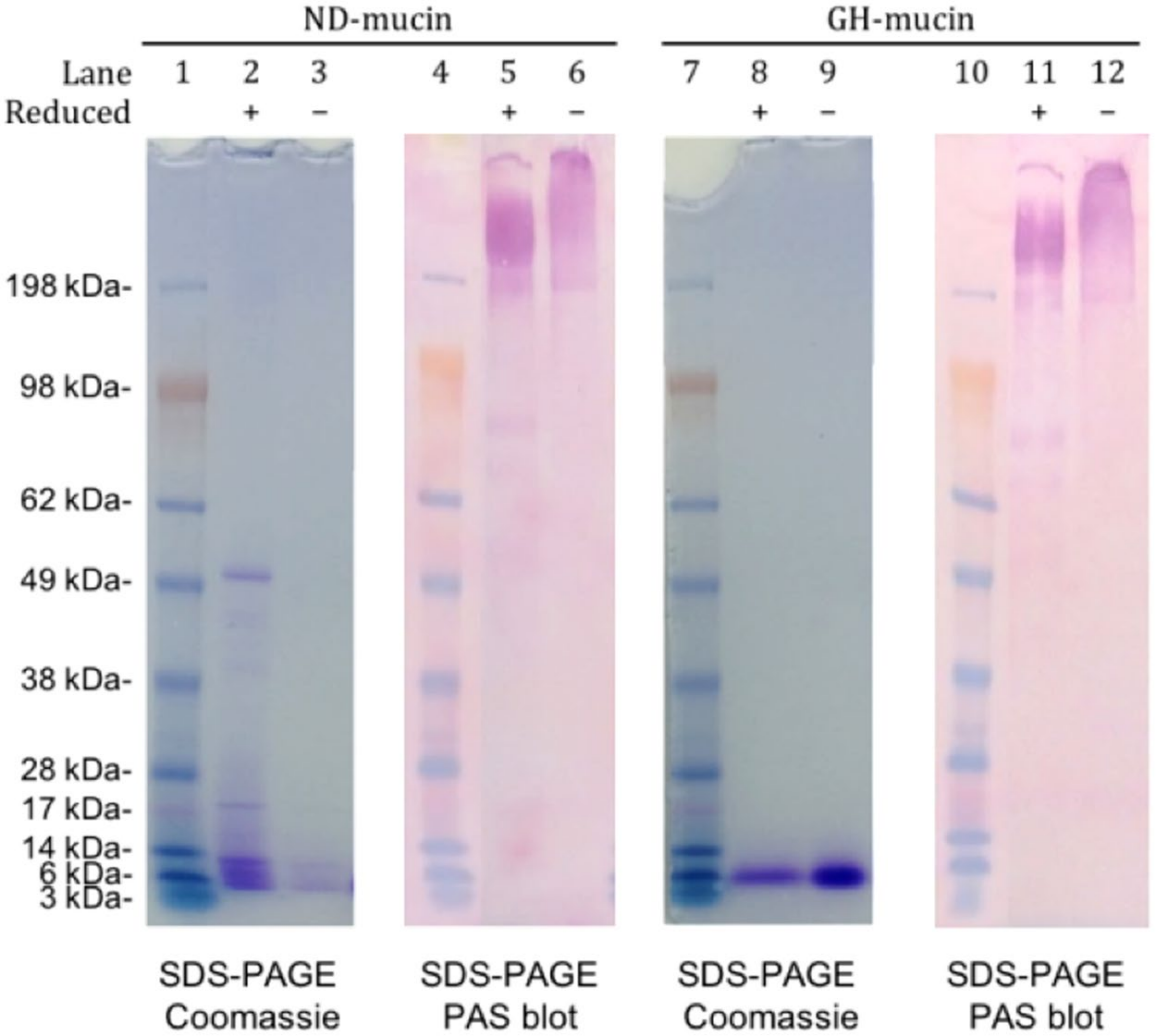
SDS-PAGE of ND- and GH-mucin preparations of porcine intestinal mucin after two-step CsCl isopycnic density gradient centrifugation. Electrophoresis was carried out using 4–12% NuPAGE Bis-Tris gel and blotted onto nitrocellulose membrane, images from the figure were cropped from different parts of the same gel with blotting performed with the same set of materials (see Supplementary Figure S11). ND- and GH-mucin preparations were compared with and without the inclusion of a reducing agent (DTT). Coomassie Blue stain was used to detect non-mucin proteins and PAS staining on nitrocellulose was used to detect glycoproteins. 30 μg of lyophilized mucin preparations were loaded in each lane. Molecular marker (lanes: 1, 4, 7, 10), reduced ND-mucin (lanes: 2 and 5), non-reduced ND-mucin (lanes: 3 and 6), reduced GH-mucin (lanes: 8 and 11) and non-reduced GH-mucin (lanes 9 and 12).

SDS-PAGE analysis (Fig. 1, Lanes 2 and 3) revealed ND-mucin contains considerable amounts of non-mucin proteins persisted through the non-denaturing CsCl isolation step prior being released after reduction of disulfide bonds (DTT). Due to extensive dilution of original mucus, the majority of non-mucin proteins must be associated with the mucin glycoproteins via chemical or strong physical bonds, as opposed to being physically entrapped in the unreduced mucin network. Compared to ND-mucin, GH-mucin contained very low amounts of non-mucin proteins associated with the mucin glycoprotein (see Fig. 1, Lanes 8 and 9, and Table 1). Small molecular weight components (<6 kDa) were present in both preparations, and were also observed during the CsCl centrifugation of ND-mucin (Supplementary Figure S2). Nitrocellulose blots of SDS-PAGE and agarose gel electrophoresis (0.7% w/w) showed the presence of PAS-reactive carbohydrates that is consistent with the migration pattern of mucins (Fig. 1 and Supplementary Figure S3). Some faint bands detected in DTT treated samples (Fig. 1, Lanes 5 and 6) around ∼ 200 and 90 kDa Mw mark could be attributed to some glycosylated immunoglobulins and their fragments. Table 1 provides mucin quantification data of ND- and GH-mucin preparations, highlighting that non-mucin components are a minor fraction of the ND-preparation (ca. 7%) and virtually absent in GH-mucin.

**Table 1:** Summary and yield of ND- and GH-porcine intestinal mucin preparations after two-step isopycnic density gradient centrifugation.

Preparation	Total protein (μg)	Mucin (μg)	Protein (μg)	Protein		Net Muc2 yield (%)
				Gross (%)	Net (%)	
Native	10	9.27	1.61	16.1	11.29	92.68
Denatured	10	9.88	0.86	8.56	3.75	98.83
Denatured (diafiltered)	10	10	0.48	4.8	0	100

A PAS-microtitre assay was used to determine quantitatively the glycoprotein concentration using diafiltered GH-mucin preparation as a standard. Results shown represent the mean of analytical triplicates.

### Microrheological behavior of ND-mucin solutions

**Figure 2:**
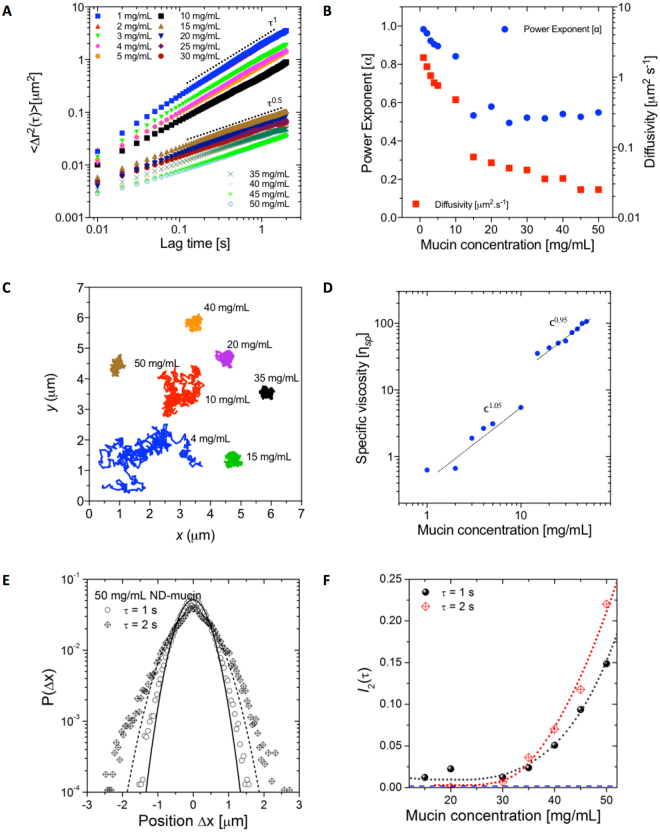
Microrheological characterization of ND-mucin solutions. (**A**) The ensemble averaged MSD curves of 0.5 μm colloidal particles in 1 ≤ c ≤ 50 mg/mL ND-mucin. Two distinct regimes were observed in ND-mucin based on the power law of the MSD curves. The trend lines show the log-log scaling of τ^1^ and τ^0.5^ as guides to highlight the transition from diffusive behavior in solutions up to 10 mg/mL, to sub-diffusive for concentrations 15 mg/mL and above. (**B**) The dependence of power law exponents, α (left Y axis), and diffusivity coefficients, *M* (right Y axis), for 1 ≤ c ≤ 50 mg/mL ND-mucin. A transition is observed between 10 and 15 mg/mL where the power exponent becomes independent and diffusivity decreases with increasing concentration. (**C**) Representative trajectories of individual 0.5 μm fluorescent colloidal particles embedded within mucin solutions of increasing concentration. (**D**) Specific viscosity (${\eta }_{sp}$) scaling of 1 ≤ c ≤ 50 mg/mL ND-mucin. (**E**) Comparison of probability distributions of 0.5 μm colloidal particles movement in Δ*x*-distances in a lag time of 1 s and 2 s for 50 mg/mL ND-mucin solution. The solid and dashed lines are Gaussian distributions for 1 s and 2 s, respectively fitted to the data. For lag time of 2 s, the deviation from Gaussian distribution is observed in the increased deviations in the tail regions, as well as the increased probability in the area around the maximum. (**F**) The excess kurtosis parameter *I*_2_(τ) plotted against mucin concentration for the lag times of 1 s and 2 s (dotted lines are parabolic spline to the data to guide the eye). For τ = 1 s and 2 s the degree of deviation was not measurable below concentrations of 10 and 20 mg/mL, respectively. A dashed line indicates *I*_2_(τ) for water.

In order to study the rheological properties of Muc2 mucin preparations on the microscale we have utilized PTM technique, which probes the thermally driven (diffusive) motion of sub-micrometer sized particles. Figure 2A shows the ensemble averaged mean square displacement (MSD) curves for ND-mucin across a range of concentrations 1 ≤ *c* ≤ 50 mg/mL. For each concentration, the power law remains constant across a span of time-lags between 10 and 2000 milliseconds. An abrupt transition was observed at a physiologically relevant concentration between 10 and 15 mg/mL where the diffusivity (*M*) and power law exponent (α) show a sharp decrease (Fig. 2B). Notably, this transition sees tracer particle motion changing from diffusive (α ∼ 1) to sub-diffusive (α ∼ 0.5). Figure 2C illustrates this transition by showing trajectories of individual tracer particles, and how trajectories’ shape changes with concentration. At concentrations above the transition (c ≥ 15 mg/mL), the diffusivity steadily decreases, while the power parameter, α, remains roughly constant at 0.5, suggesting that viscoelastic behavior of the system defined by the ratio $G^{\prime\prime} /G^{\prime} =\,\tan \,\delta =\,\tan (\frac{1}{2}\pi \alpha )$ remains unchanged once in a sub-diffusive state. For MSD spectra where α ∼ 1, the diffusivity is inversely proportional to the solution viscosity via the Stokes-Einstein equation (see Eq. 4). For the concentrations with sub-diffusive particle motion (α < 1), the viscosity depends on frequency, and according to Eq S3 and Eq S4 for α ∼ 0.5 G′(ω) ≈ G″(ω). This circumstance enables us to define the effective viscosity using the expression for ${\eta }^{\ast }$ (Eq S5) as ${\eta }_{{\rm{eff}}}^{\ast }={\eta }^{\ast }({\omega }^{\ast })$ where the value of frequency ${\omega }^{\ast }=\frac{1}{{t}^{\ast }}={({\rm{\Gamma }}(1+\alpha ))}^{1/\alpha -1}$ (for α = 0.5 ω* ∼ 1.27 Hz and t* ∼ 0.7854 s) (See Supplementary Section 4). At the frequency ω*, the form of the expression for ${\eta }^{\ast }$coincides with the classic Stokes-Einstein equation (Eq. S2), enabling consistent comparison between *η* for c ≤ 10 mg/mL and ${\eta }^{\ast }$ for c ≥ 15 mg/mL.

The dependency of the specific viscosity $({\eta }_{{\rm{sp}}}=\frac{\eta }{{\eta }_{s}}-1)$ on mucin concentration shows an order of magnitude abrupt increase around 15 mg/mL (Fig. 2D), which correlates with the change in diffusivity and solution viscoelasticity (Fig. 2B). The scaling of specific viscosity with concentration is found to be linear in both concentration ranges, i.e. *c* ≤ 10 mg/mL and *c* ≥ 15 mg/mL. This behavior is in contrast to that observed previously for purified mucins^5,27^, where low concentrations showed Fuoss law scaling ${\eta }_{{\rm{sp}}} \sim {c}^{\frac{1}{2}}$, and, at higher concentrations, a transition to an entangled regime with the scaling exponent of specific viscosity exceeding ^3^/_2_ was observed. Further analysis of ND-mucin rheological behavior was undertaken by employing the concept of intrinsic viscosity. In general, intrinsic viscosity, $[\eta ]$, is a physical measure that characterizes the volume of a polymer chain in solution and is proportional to the hydrodynamic radius and shape of the molecule. The detailed analysis presented in Supplementary Section 5 corroborates that the linear scaling of ${\eta }_{{\rm{sp}}}$ with concentration points to the presence of complex mucin oligomers that rheologically behave as “particles”. The presence of oligomers is further inferred from SDS-PAGE analysis (Fig. 1, Lanes 5 and 6), which shows the reduction in the effective molecular weight of the mucin band upon treatment with disulfide bond reducing agent, DTT^17^. For *c* ≥ 15 mg/mL, the linear scaling of the specific viscosity with concentration is consistent with molecular assemblies of dense viscoelastic gels^28^.

### Microrheological heterogeneity of ND-mucin solutions

The heterogeneity of rheological properties of mucus systems is well-documented^29^. For *in vivo* or *ex vivo* mucus, the rheological heterogeneity can be associated with the heterogeneous composition (e.g. different mucin genetic types or different glycoforms) as well as imperfect mixing of material extruded from different secretory granules by goblet cells lining the epithelial surface. For purified mucins, the uncovered heterogeneity may originate from the nucleation driven mechanism of network formation^30^. One of the useful approaches for evaluating heterogeneity based on microrheological data is the analysis of the van Hove distribution of particle displacements^31^, $P({\rm{\Delta }}x=x(t+\tau )-x(t))$, which ascertains probability of particles displacement within a certain lag time, *τ*. In structurally homogenous materials, where all particles are in the same micro-environment, the van Hove distribution is Gaussian, while in complex materials the deviation from the Gaussian distribution indicates the presence of heterogeneities; i.e., different tracer particles probe different regions of the fluid with distinct rheological properties. A parameter that characterizes the degree of deviation is called excess kurtosis, ${I}_{2}(\tau )$ (see Supplementary Section 6), which is a measure of the ‘tail’ of the probability distribution^31^ (for the Gaussian distribution ${I}_{2}(\tau )=0$).

**Figure 3:**
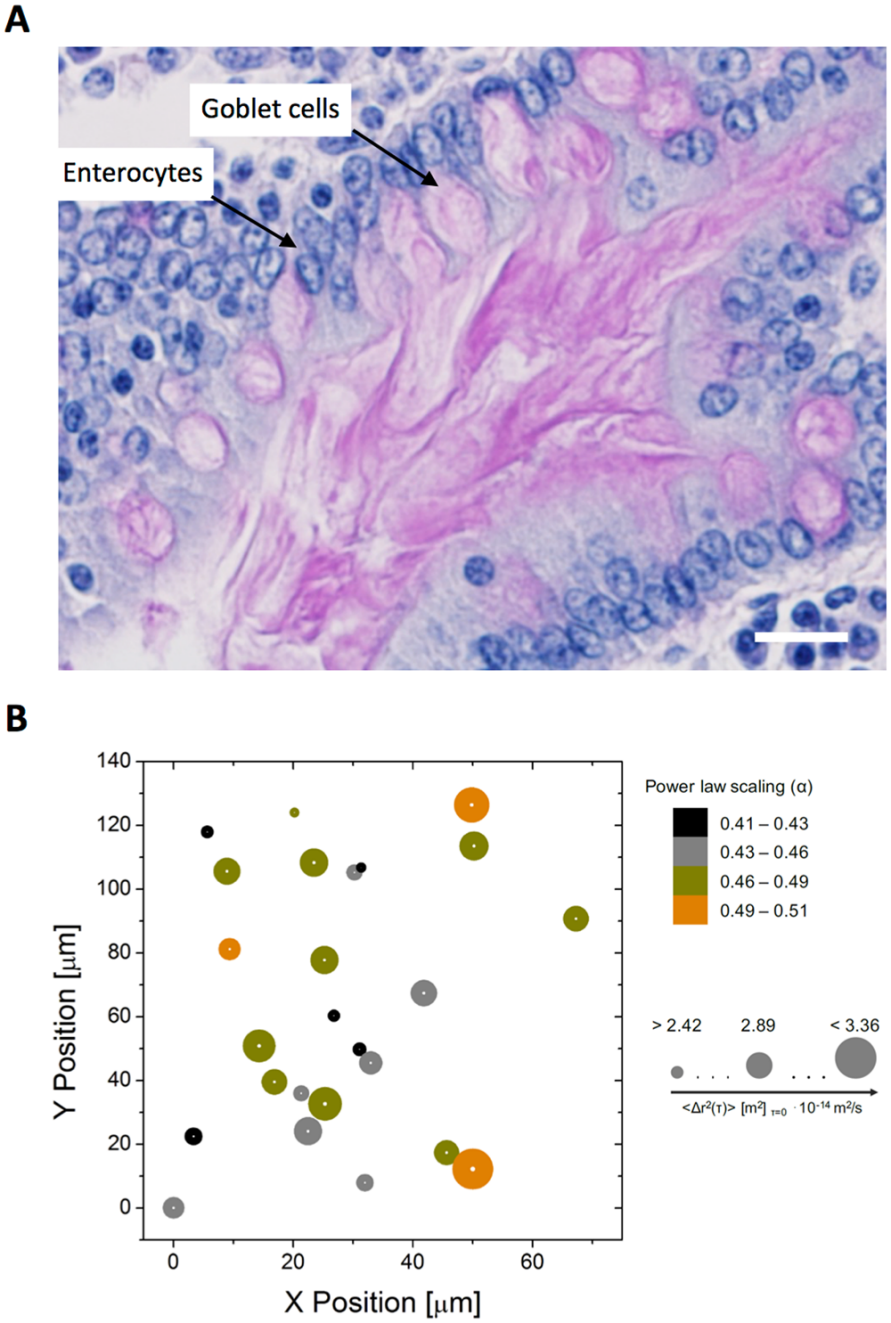
A structural model of mucus comprising rheologically heterogeneous domains blended together. (**A**) Periodic acid-Schiff staining of the pseudostratified columnar epithelium from the proximal small intestinal porcine mucosa which illustrates the presence of domains in physiological mucus. Note the pink staining of mucus from goblet cells into the intestinal lumen and the blue staining of nuclei. Scale bar 20 μm. (**B**) Visualization of the rheological heterogeneity, X- and Y-positions, of movement of individual 0.5 μm colloidal particles in 50 mg/mL ND-mucin solution. The size of circles indicates diffusivity and the color corresponds to the power law scaling of the MSD spectra.

Figure 2E shows the van Hove distribution of particles displacements in 50 mg/mL ND-mucin at a lag time of *τ* = 1 s and *τ* = 2 s. The deviation from the Gaussian distribution is clearly observable, with the tendency of ${I}_{2}(\tau )$ values to increase with increasing the lag time. This suggests that with increasing time, the ensemble of tracer particle probes the progressively larger space, resulting in the more pronounced contribution from rheological heterogeneities. To illustrate the concentration dependence of rheological heterogeneity in ND-mucin, the dependency of the excess kurtosis of the van Hove distribution, ${I}_{2}(\tau )$, depending on mucin concentration is quantified for a broad range of concentrations, $1\le c\le 50$ mg/mL, and is shown in Fig. 2F. (The original van Hove distributions used to calculate ${I}_{2}(\tau )$ for a lag time of *τ* = 1 s and *τ* = 2 s are shown in Supplementary Figures S5A and S5B, respectively). The results show that mucin solutions are relatively homogenous $({I}_{2}(\tau =2\,s) < 0.25)$ if compared to other gelling systems, such as acrylate Carbopol® where values of ${I}_{2}(\tau )\cong 4$ have been reported^32^. Despite the relatively low level of excess kurtosis, the heterogeneity is still significant, especially at the highest mucin concentrations. This clearly identifies the presence of micro-domains with varying viscoelastic properties. We also note that for *τ* = 2 s the van Hove distributions also feature an increased probability around Δ*x* = 0 (Supplementary Figure S5B), this increase is due to the higher than average level of covariance, $\langle ({\rm{\Delta }}x-\langle {\rm{\Delta }}x\rangle )({\rm{\Delta }}y-\langle {\rm{\Delta }}y\rangle )\rangle$. Effectively, if a particle has displacement Δ*x* ≈ 0 then there is a higher than average chance that the particle also has displacement Δy ≈ 0. This behavior indicates that a significant number of particles show limited mobility, which provides further evidence of heterogeneous distribution of rheological properties at the micrometer scale. The increased covariance at Δ*x* = Δ*y* = 0, prompted a further probe into the details of heterogeneity by evaluating the parameters of the caged diffusion, where we follow the theoretical framework by Doliwa and Heuer^33^. This approach has been successfully utilized to characterize caged-diffusion in other systems^34,35^, and is based on correlating two successive displacements of equal lag time. For the caged diffusion, the average distance of the second displacement ${\tilde{{\boldsymbol{r}}}}_{12}^{x}$ is negative and scales linearly with the distance of the preceding displacement, ${{\boldsymbol{r}}}_{01}$,

1 $$\langle {\tilde{{\boldsymbol{r}}}}_{12}^{x}\rangle =\langle \frac{{{\boldsymbol{r}}}_{01}\cdot {{\boldsymbol{r}}}_{12}}{|{{\boldsymbol{r}}}_{01}|}\rangle =-\beta {{\boldsymbol{r}}}_{01}$$

where *β* is a positive constant. If *β* is zero then the particle motion is random, and hence non-caged. Supplementary Figure S5C and D show a clear absence of any correlation between $\langle {\tilde{{\boldsymbol{r}}}}_{12}^{x}\rangle$ and ${{\boldsymbol{r}}}_{01}$ at lag times of 1 and 2 s, respectively. This result is very significant, as it provides strong evidence against biphasic or microgel-like structure of mucin solutions. The presence of heterogeneities and absences of clear domain boundaries (*β* ∼ 0) leads us to propose a structural model in which rheologically (and potentially compositionally) heterogeneous domains are blended together without clearly defined interfaces. This model is largely inspired by the microstructure of the native mucosa as can be seen from the histological micrographs of the small intestine presented in Fig. 3A. To illustrate the proposed model, Fig. 3B presents a map of rheological properties probed by the tracer particles across an area of 75 × 140 μm. The variations in rheological properties are captured in the changes of diffusivity (*M*; size of circles) and power exponent (*α*; color map) across the probed area.

### Micromechanical and macroscale rheological behavior of ND-mucin solutions: on the emergence of yield stress behavior and comparison with the rheology of crude intestinal mucus

**Figure 4:**
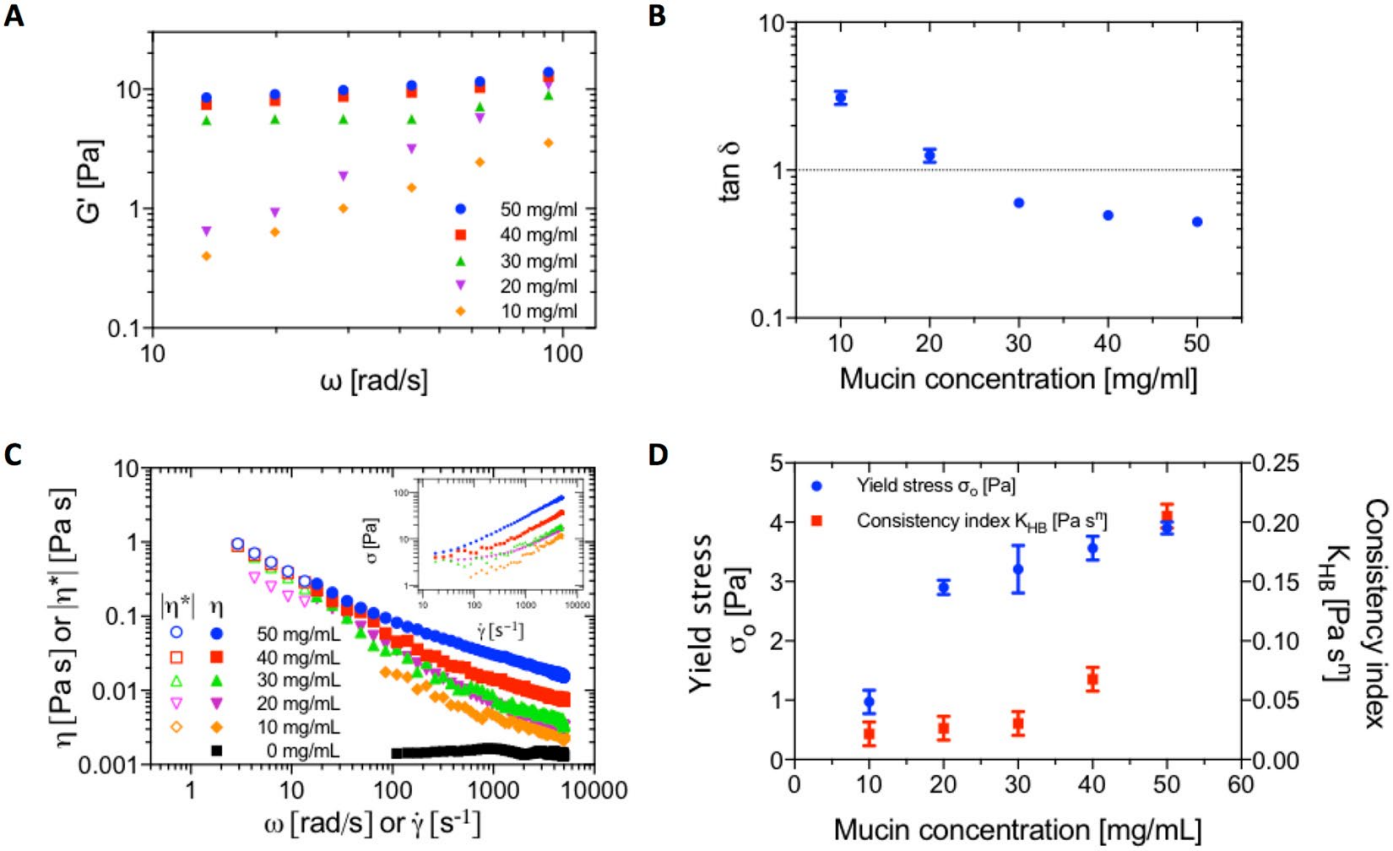
Rheological behavior of ND-mucin solutions. (**A**) Comparison of the storage moduli, G′ (ω) of 10 ≤ c ≤ 50 mg/mL ND-mucin. For concentrations below 10 mg/mL, the oscillatory spectra were within the standard error from that of pure buffer. (**B**) The loss tangent (tan δ), a ratio of the loss modulus (G″) to storage modulus (G′) of 10 ≤ c ≤ 50 mg/mL ND-mucin. (**C**) Steady shear viscosity, η, and complex viscosity, |η*|, of 10 ≤ c ≤ 50 mg/mL ND-mucin versus shear rate, $\dot{\gamma }$ and angular frequency, ω, measured using parallel-plate geometry at a gap of 40 μm. Insert shows the plot of shear stress, σ, versus shear rate, $\dot{\gamma }$, of the same data. (**D**) Parameters of the Herschel-Bulkley equation; yield stress σ_0_ and consistency index *K*_HB_, evaluated as best fits to the shear stress, σ, versus shear rate, $\dot{\gamma }$, of 10 ≤ c ≤ 50 mg/mL ND-mucin. The flow index, *n*, had a mean value of 0.73 (SD ± 0.02).

To probe the nature of the transition from diffusive to sub-diffusive motion, we have performed complimentary small amplitude oscillatory shear (SAOS) measurements to confirm the formation of a gel-like fluid in concentrated ND-mucin solutions. The oscillatory shear spectra of ND-mucin solutions show no overlap between G′ and G″ above 20 mg/mL (Supplementary Figure S6) and the dependency of G′ on ω $(G^{\prime}  \sim {\omega }^{n})$ is weak as shown in Fig. 4A, with scaling exponents *n* < 0.2, which is a characteristic of weak ‘flowable’ gels. The concentration induced transition observed in microrheological measurements is also captured in SAOS spectra as shown in Fig. 4B, where at concentration ∼ 20 mg/mL, the viscous dominated $(\tan \,\delta  > 1)$ mechanical response transitions to an elastic dominated one $(\tan \,\delta  < 1)$. It is evident the sub-diffusive character of particle motion on the micrometer scale coincides with the gel-like behavior in the bulk. However, an important distinction has to be made: at the micrometer-scale domain $\tan \,\delta \approx 1$, while bulk samples in the concentration range 30 ≤ *c* ≤ 50 mg/mL show $\tan \,\delta \approx 0.5$. This apparent discrepancy, however, provides strong evidence that at high concentrations the mucus microstructure is comprised of microscale domains fused together as illustrated in Fig. 3. While each domain may remain relatively fluid $(\tan \,\delta \approx 1)$, their assembly is in a jammed state due to limited exchange and rearrangement of molecules between the neighboring micro-domains.

To further test our hypothesis, we have performed narrow gap high shear rotational rheometry and applied the Cox-Merz rule to correlate the SAOS data with that obtained from the steady shear experiments. The Cox-Merz rule overlay plots show the steady shear and SAOS complex viscosities mapped against shear rate ($\dot{\gamma }$; filled symbols) or angular oscillation frequency (ω; hollow symbols) for a series of concentrated solutions of ND-mucin (Fig. 4C). The $\sigma =f(\dot{\gamma })$ curves clearly show that the flow behavior of ND-mucin is characterized by an apparent yield stress that dominates the low shear rate region (Fig. 4C, inset). The flow curves can be successfully modelled using the Herschel–Bulkley equation with a flow index value of 0.73 (Eq. S1)^36^. The concentration induced transition observed in PTM and SAOS experiments also finds its manifestation in the values of the apparent yield stress; for ND-mucin the transition in the yield stress is observed between 10 and 20 mg/ml (Fig. 4D). Evidently, the increase in concentration induces the assembly of ND-mucin and results in the formation of a weak assembly that exists only at low shear stresses (<3 Pa).

The observed behavior of the bulk ND-mucin is similar to microgel systems or ‘shear’ gel suspensions, such as Carbopol hydrogels^35^ and agarose ‘fluid’ gels^37^, respectively. The proposed model of micro-domains ‘swirled’ together but without distinct boundaries (Fig. 3) fits well with the experiment; within this framework, the yield stress and ‘gel-like’ viscoelasticity $(\tan \,\delta  < 1)$ stem from its micro-domain assembly, while within the micro-domains, probed by PTM, the viscoelastic behavior is more ‘fluid-like’ $(\tan \,\delta \approx 1)$.

The macroscale rheological properties of concentrated ND-mucin solutions correspond well to that of the native mucus preparations, which were measured immediately after being removed from the proximal small intestine. As shown in Supplementary Figure S7, small intestinal mucus is characterized by a similar set of rheological properties found previously using the purified ND-mucin model at concentrations ≥20 mg/mL, these include shear thinning (Supplementary Figure S7A), yield stress behavior (Supplementary Figure S7B), and G′ dominated viscoelastic response (Supplementary Figures S7C and D). This set of properties, we hypothesize, may be advantageous for the physiological function of mucus, whereby yield stress and viscoelastic response are key for integrity of the mucus layer, while an apparent mechanical fragility manifested in the low values of the yield stress and shear thinning appear to be key for mucus extrusion from secretory granules, and its subsequent spreading and turnover.

### Probing physicochemical and biochemical principles of mucin assembly leading to the formation of a mucus-like network

The presented multi-scale analysis of rheological properties of mucin prompts two key hypotheses to test. First, we aim to evaluate the nature of mucin-mucin interactions responsible for the formation of viscoelastic fluids; in particular we seek to evaluate such factors as non-mucin components, hydrogen bonding, disulfide bridges and Ca^2+^-mediated links, as well as pH. Second, we aim to evaluate the process of breakdown of mucin associative network under high shear conditions, and assess whether this network dis-assembles down to the constituent mucin monomers, or alternatively blocks of several mucin sub-assemblies/oligomers.

#### The effect of non-mucin components

The non-mucin components appear to affect all aspects of rheological behavior from dilute solutions to the formation of an associative network. Devoid of the majority of non-mucin components, GH-mucin preparations exhibit Fuoss law scaling of specific viscosity $({\eta }_{{\rm{sp}}} \sim {c}^{\frac{1}{2}})$ indicating formation of semi-dilute polymer solutions (Supplementary Figure S8A), diffusive scaling (α ∼ 1) of tracer microparticle MSD (Supplementary Figure S8A, inset), absence of yield stress behavior (Supplementary Figure S8B, inset), 10-times lower values of G′ compared to ND-mucin (Supplementary Figure S8C), and G″ dominated viscoelastic response $(\tan \,\delta  > 1)$ (Supplementary Figure S8D), making GH-mucin rheological behavior consistent with that of a weakly-associative polymer. We also note the absence of the critical transition at concentrations observed in ND-mucin. All these elements point towards a critical role of non-mucin components in the formation of the associative network.

Further, the involvement of non-mucin components in the formation of mucin oligomers is inferred from SDS-PAGE analysis. As shown in Fig. 1, the large number of non-mucin proteins is released upon treatment with disulfide bond reducing agent, DTT, which is capable of breaking down the oligomers. The oligomer breakdown is evident from the reduction in the effective molecular weight of the mucin band upon treatment with DTT^12^. This results indicates that non-mucin proteins are either covalently linked to mucin via disulfide bonds or their binding is dependent on the entanglement of mucin formed by inter- or intra-molecular disulfide bonds. Previous work has established that pure mucin (i.e. void of non-mucin components) is capable of higher order assembly^38,39^; here we show that in addition to the innate property of mucin to form self-assembled structures, the non-mucin components are instrumental for stabilising physical crosslinks between mucin domains, and potentially responsible for promoting formation of additional links that facilitate molecular assembly.

Evidence for the role of non-mucin components can be gained by analyzing the shear thinning behavior of both ND- and GH-mucin. In ND-mucin, the values of the consistency index (*K*_HB_) increase with concentration while the values of the yield stress (σ_0_) remain unchanged for *c* ≥ 20 mg/mL (Fig. 4D). The increase in *K*_HB_ is consistent with the increasing degree of crosslinking, which is expected to occur with the increasing mucin concentration. Here we should distinguish between physical crosslinks such as entanglements and other types of crosslinks associated with hydrogen bonding and disulfide bridges. Since *K*_HB_ and σ_0_ do not correlate, the majority of crosslinks must be in a form of physical entanglements that provide little contribution to the formation of the associative network that confers ND-mucin its viscoelastic response and yield stress behavior. Upon yielding, with increasing $\dot{\gamma }$, ND-mucin solutions display shear thinning behavior (*n* = 0.73). As a result of shear thinning, the viscosity of ND-mucin solutions at high shear rates appears to be lower than that measured using PTM and lower than GH-mucin of similar concentration (cf. Fig. 4C and Supplementary Figure S8B). The effect is particularly striking for lower concentrations of ND-mucin; as can be seen in Fig. 4C, the effective viscosity of 10 mg/mL ND mucin is not too dissimilar from pure solvent at $\dot{\gamma } \sim {\mathscr{O}}({10}^{3}\,{s}^{-1})$. This indicates a reduced phase volume of the mucin after shear-induced structural breakdown^40^.

The character of network breakdown suggests that a range of interactions of different strengths is responsible for the formation of an associative network in ND-mucin. Upon shearing, the weaker links may break, while stronger ones are more likely to endure. The high shear behavior of mucin solutions can be analyzed in terms of the effective hydrodynamic volume. Although such analysis does not enable calculation of the true hydrodynamic volume, ${[\eta ]}_{c\to o}$, determining ${[\eta ]}_{\dot{\gamma }\to \infty }^{\ast }$ is a useful comparative tool to ascertain changes in effective hydrodynamic size of polymer species produced by the action of shear. Supplementary Table S3 summarizes values of ${[\eta ]}_{\dot{\gamma }\to 4000\,{s}^{-1}}^{\ast }$ defined as ${\eta }_{\dot{\gamma }}={\eta }_{s}(c{[\eta ]}_{\dot{\gamma }}^{\ast }+1)$. One can see that values ${[\eta ]}_{\dot{\gamma }\to 4000\,{s}^{-1}}^{\ast }$ for ND-mucin are up to 3 times lower compared to that of GH-mucin. This suggests that aggregation and/or formation of mucin oligomers in ND-mucin results in the lower phase volume of mucin phase compared to a ‘molecular’-like polymer solution formed by GH-mucin. With increasing concentration, the difference between ND- and GH-mucin lessens, which indicates a lower degree of fragmentation upon shear which is consistent with the formation of a more resilient network.

The presence of non-mucin components can enhance the complexity of mucin supramolecular complexes. Typically mucins form dimers, which require further association to form a network during biosynthesis^11^. In the case of intestinal mucus, it has been proposed that Muc2 forms trimers via N-termini^41^. Our findings support the concept that oligomers can be formed through stable links facilitated by non-mucin components. More importantly, such oligomers can have more complex structures, including highly-branched ones. The assembly of such molecular ‘LEGO’ blocks may be crucial for the formation of a 3D network and its reorganization. Under this scenario, reorganization of the network requires rearrangement of a small number of cross-links for mucin to flow or reverse back into a gel-like state. Such adaptability, we hypothesize, can explain a number of key mucin functionalities associated with its barrier function, lubrication, and turnover.

#### The effect of disulfide bridges, hydrogen bonds, and Ca^2+^ mediated links

**Figure 5:**
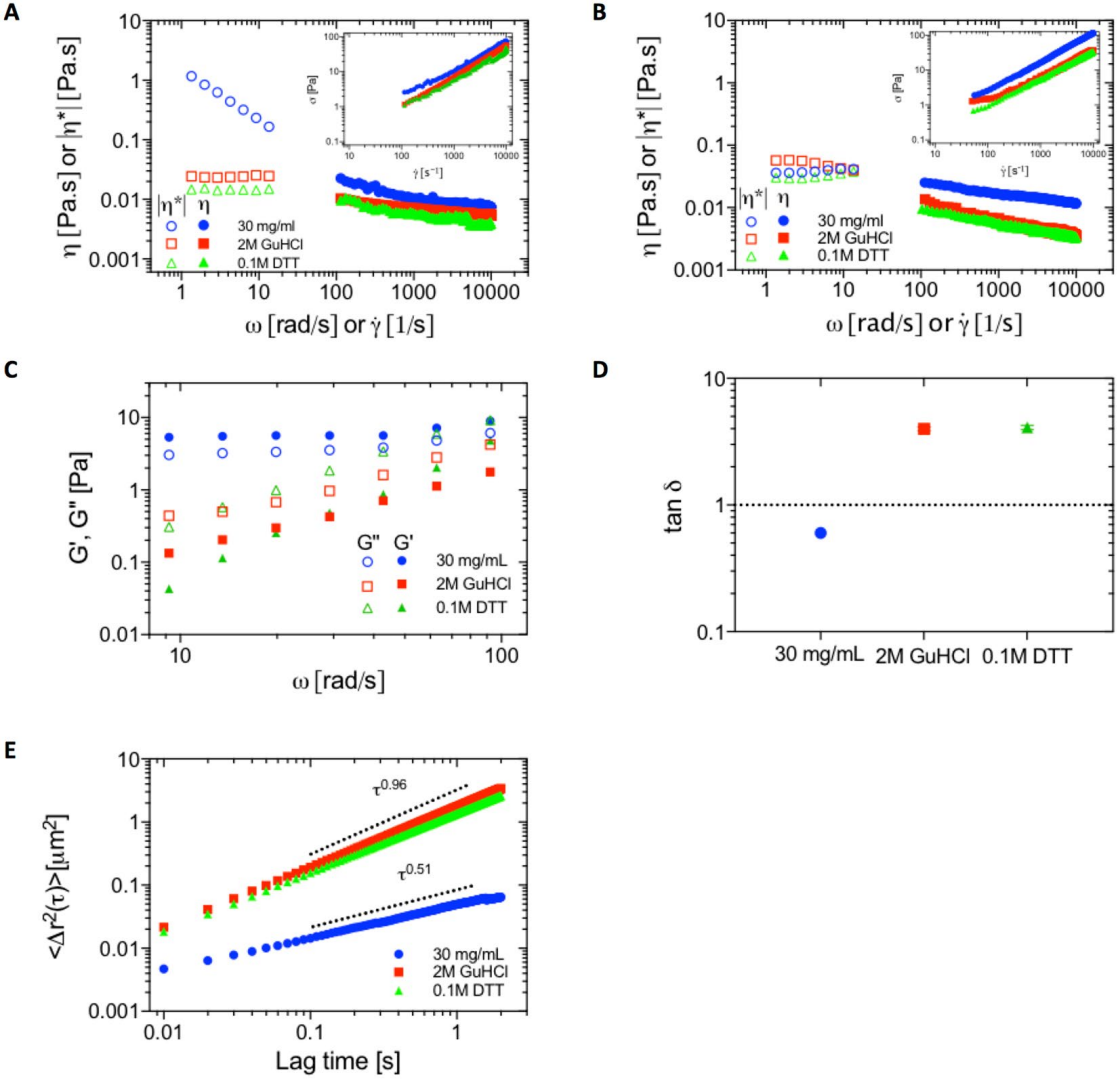
The effect of denaturing agents on rheological properties of ND- and GH-mucin solutions. (**A**) Steady shear viscosity, η, and oscillatory viscosity, |η*|, of 30 mg/mL ND-mucin treated with 0.1 M DTT and 2 M GuHCl versus shear rate, $\dot{\gamma }$, and angular frequency, ω, measured using parallel-plate geometry at a gap of 40 μm. Insert shows the plot of shear stress, σ, versus shear rate, $\dot{\gamma }$, of the same data. (**B**) Steady shear viscosity, η, and oscillatory viscosity, |η*|, of 30 mg/mL GH-mucin treated with 0.1 M DTT and 2 M GuHCl versus shear rate, $\dot{\gamma }$, and angular frequency, ω, measured using parallel-plate geometry at a gap of 40 μm. Insert shows the plot of shear stress, σ, versus shear rate, $\dot{\gamma }$, of the same data. (**C**) Comparison of the storage modulus, G′ (ω; solid symbols), and loss modulus, G″ (ω; empty symbols), of 30 mg/mL ND-mucin treated with 0.1 M DTT and 2 M GuHCl. D.) The loss tangent (tan δ), the ratio of loss modulus (G″) to storage modulus (G′), of 30 mg/mL ND-mucin treated with 0.1 M DTT and 2 M GuHCl. E.) The ensemble averaged MSD curves of 0.5 μm colloidal particles in 30 mg/mL ND-mucin treated with 0.1 M DTT and 2 M GuHCl with the log-log scaling to be τ^0.51^ τ^0.96^ and τ^0.98^, respectively.

Here, we elaborate on the nature of the cross-links and provide further proof that the mucin assembly is facilitated by non-mucin components. Upon addition of 0.1 M DTT or 2 M GuHCl to the buffer, striking changes in the elastic response and the yielding behavior are observed. DTT and GuHCl result in a complete abatement of the yield stress behavior in ND-mucin as shown in Fig. 5A; this result is a clear demonstration of the connection between the yield stress and the formation of the associative network. Compared to the case of ND-mucin, changes induced by GuHCl and DTT in GH-mucin are less dramatic; both have little impact on oscillatory shear spectra and microrheological properties as shown in Fig. 5B.

Upon addition of either DTT or GuHCl to ND-mucin, we observe a marked reduction in G′ (at least one order of magnitude, Fig. 5C; filled symbols), transformation of a gel-like response $(\tan \,\delta  < 1)$ to a fluid-like response $(\tan \,\delta  > 1)$ (Fig. 5D), dramatic decrease of the effective network relaxation time, as shown by the scaling of G″ with oscillatory frequency (Fig. 5C), as well as increased mobility of tracer particles and their purely diffusive (α ∼ 1) behavior (Fig. 5E). Both treatments induce very similar changes in rheological behavior, indicating that disruption of the associative network – regardless of physical or chemical mechanism – results in similar properties of the ‘end-product’. While profound changes are expected for DTT, the fact that 2 M GuHCl results in similar changes illustrates the importance of hydrogen bonds and protein folding in the formation of the mucin network.

Two differences between GuHCl and DTT can be noted: first, we observe an increase in the power law exponent of G″ on angular frequency from 1 to 1.5 for GuHCl and DTT treatments, respectively (Fig. 5C); for reference, the power law exponent is 0.3 in buffer. Second, DTT results in lower high shear viscosity (${\eta }_{\dot{\gamma }=4000{s}^{-1}}=5.1$ [mPa·s]) compared to GuHCl (6.0 [mPa·s]). These differences suggest that DTT may result in a more profound disruption of the network compared to GuHCl, which is consistent with the model where DTT would cleave disulfide bonds and result in complete dissociation of elementary mucin oligomers into the individual mucin molecules. As a secondary mechanism of network disruption, we suggest that DTT may weaken hydrogen bonding by denaturing the highly structured mucin C- and N-termini formed by extensive disulfide bonding, as well as cleaving disulfide bonds in non-mucin proteins.

As mentioned above, GuHCl and DTT have little effect on oscillatory shear spectra and microrheological properties of GH-mucin. However some changes should be noted; both denaturing agents influence the shear thinning behavior as shown in Fig. 5B, which indicates the disruption of a weak associative network in GH-mucin. A small decrease in high shear viscosity in DTT (${\eta }_{\dot{\gamma }=4000{s}^{-1}}=4.0$ [mPa·s]) compared to GuHCl (4.6 [mPa·s]) may indicate an effect related to the reductive dissociation of constituent oligomers (i.e., a reduction in the effective molecular weight of polymeric species).

We also note that the effect of DTT and GuHCl on shear thinning is more significant for GH-mucin compared to ND-mucin. While it may appear counterintuitive, this can be rationalized in the context of whether the behavior is dominated by polymers or gel-like microparticles. For GH-mucin, which at 30 mg/mL is a semi-dilute polymer solution, the decrease in the viscosity is the result of the transition from an entangled to a less viscous dilute network. Evidently, this transition is associated with a reduction in the effective molecular weight of constituent polymers, dominated by dissociation of oligomers to mucin monomers. By contrast, ND-mucin shear thinning behavior is governed by the disruption of the micro-domain network that results in the formation of shear-gel particles, which qualitatively behave as a suspension with viscosity much lower compared to the polymer solution.

**Figure 6:**
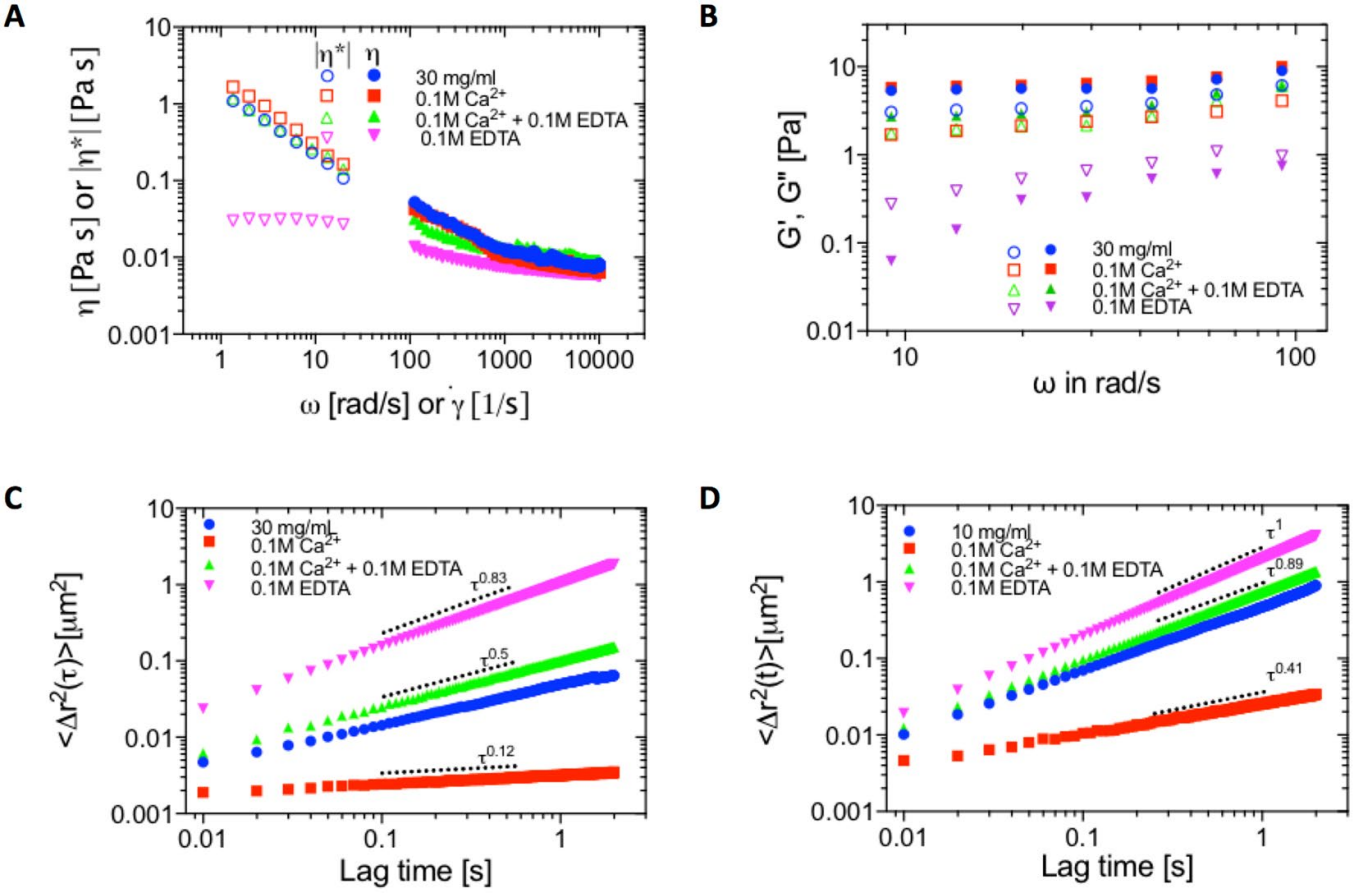
The effect of Ca^2+^ and Ca^2+^ chelator (EDTA) on rheological properties of ND-mucin solutions. (**A**) Steady shear viscosity, η, and oscillatory viscosity, |η*|, of 30 mg/mL ND-mucin treated with 0.1 M Ca^2+^, 0.1 M Ca^2+^/0.1 M EDTA and 0.1 M EDTA versus shear rate, $\dot{\gamma }$, and angular frequency, ω, measured using parallel-plate geometry at a gap of 40 μm. Insert shows the plot of shear stress, σ, versus shear rate, $\dot{\gamma }$, of the same data. (**B**) Comparison of the storage modulus, G′ (ω), and loss modulus, G″ (ω), of 30 mg/mL ND-mucin treated with 0.1 M Ca^2+^, 0.1 M Ca^2+^/0.1 M EDTA and 0.1 M EDTA. (**C**) The ensemble averaged MSD curves of 0.5 μm colloidal particles in 30 mg/mL ND-mucin treated with 0.1 M Ca^2+^, 0.1 M Ca^2+^/0.1 M EDTA and 0.1 M EDTA with the log-log scaling to be τ^0.46^, τ^0.12^, τ^0.5^ and τ^0.83^, respectively. (**D**) The ensemble averaged MSD curves of 0.5 μm colloidal particles in 10 mg/mL ND-mucin treated with 0.1 M Ca^2+^, 0.1 M Ca^2+^/0.1 M EDTA and 0.1 M EDTA with the log-log scaling to be τ^0.85^, τ^0.41^, τ^0.89^ and τ^1^, respectively.

The role of Ca^2+^ appears complex, but nonetheless straightforward to corroborate. For all tests we have run a ‘control’ sample which contains an equimolar mixture of Ca^2+^ and EDTA that are designed to cancel the activity of each other; across tests we saw little difference between ND-mucin and the ‘control’ sample. Figure 6 shows that the addition of chelating EDTA results in a marked decrease of the shear viscosity of 30 mg/mL ND-mucin (Fig. 6A). As expected, the addition of Ca^2+^ increases the G′ of ND-mucin and reduces the value of $\tan \,\delta$ as shown in Fig. 6B. The microrheological environment is also affected by the presence of EDTA and Ca^2+^. The MSD spectra (Fig. 6C) shows that the addition of Ca^2+^ results in the formation of a more elastic network, as indicated by a decrease in the power law exponent (α) below 0.5. The addition of EDTA, correspondingly, results in a more diffusive motion of tracer particles with the values of power law exponent (α) above 0.8.

The effect of Ca^2+^ and EDTA on more dilute ND-mucin solutions is also remarkable. Figure 6D shows the MSD spectra for 10 mg/mL ND-mucin with Ca^2+^ and EDTA. Addition of Ca^2+^ results in a marked strengthening of the network, as shown by decrease in the parameter α from marginally sub-diffusive (α > 0.8) to viscoelastic (α < 0.5). In contrast, addition of EDTA results in nearly entire loss of viscoelastic response (α ∼ 1). The presence of endogenous Ca^2+^ provides a simple explanation of this result. Indeed, endogenous Ca^2+^ can be present bound to one or several non-mucin proteins, enabling Ca^2+^ ions to be retained through purification and dialysis. In more dilute solutions, the addition of Ca^2+^ favors network formation, while in more concentrated solutions, there may be sufficient endogenous Ca^2+^ to establish a viscoelastic network. Neither EDTA nor Ca^2+^ show a marked effect on the steady shear flow behavior of GH-mucin compared to ND-mucin (Supplementary Figures S9A and S9B), providing direct evidence that non-mucin components are key for establishing Ca^2+^-dependent links. A similar result has been observed by Raynal *et al*.^18^ for saliva which is dominated by the MUC5B mucin, suggesting non-mucin proteins are key for establishing Ca^2+^-mediated inter-molecular links across a broad range of mucin-rich mucosal fluids.

An important question arises regarding the interplay between Ca^2+^-dependent links and hydrogen bonds. Both interactions contribute to the formation of an associative network with their disruption leading to a loss of viscoelastic response in ND-mucin. We propose, from the evidence below, that Ca^2+^-links are the dominant factor in network formation, with hydrogen bonds, as a kind of molecular ‘glue’, playing a critical role in facilitating the association of mucin with non-mucin proteins, including those involved in the formation of Ca^2+^-mediated links. An important consideration, we argue, is that most non-mucin proteins in ND-mucin solutions are attached to Muc2 mucin. This is evident from SDS-PAGE (Fig. 1), which shows few non-mucin components separated without addition of reducing agent (DTT). Thus, the majority of non-mucin proteins in ND-mucin solutions are in a bound rather than weakly-associated state. Upon addition of 0.1 M EDTA to ND-mucin (30 mg/mL), the Ca^2+^ ions are chelated which results in a markedly weaker viscoelastic response compared to Ca^2+^-enriched solutions. Formation of hydrogen bonds in the presence of EDTA is ***not*** inhibited, and therefore in the absence of Ca^2+^ the network is supported by hydrogen bonds only. The data suggest that hydrogen bonds in the absence of Ca^2+^ provide only a limited contribution to the rheological strength of the ND-mucin network.

The case of 10 mg/mL ND-mucin is equally illustrative. As evident from the data presented in Fig. 6D, the addition of 0.1 M Ca^2+^ to 10 mg/mL ND-mucin boosts solution viscoelasticity, which, we hypothesize, is a result of saturating non-mucin proteins with Ca^2+^ and the formation of Ca^2+^-mediated links. Prior to Ca^2+^ addition, non-mucin proteins (which are present and bound to mucin) have the unhindered ability to interact through hydrogen bonds, yet without Ca^2+^ this interaction is not sufficient to drive formation of a viscoelastic network. Our findings suggest that environments low in free calcium, such as the outer layer of mucus, may promote disassembly due to Ca^2+^ leaching and depletion. Likewise, some dietary forms of free Ca^2+^ or Ca^2+^ chelators may influence the viscoelasticity of the mucus barrier.

#### The effect of solvent acidification on formation of a mucin gel

Acid induced gelation has been reported previously^5,42^ and it was postulated that disulfide and hydrogen bonds are responsible for the assembly of mucin monomers into supramolecular aggregates that at certain concentrations may form a gel-like network. It is also important to note that substantial repulsion between mucin molecules is due to hydration shells of oligosaccharide side chains as well as electrostatic contributions from the negatively charged sialic acid residues. At lower pH, the ionisation of sialic acid is reduced and hence the repulsive barrier may diminish, paving the way to enhancement of mucin assembly.

**Figure 7:**
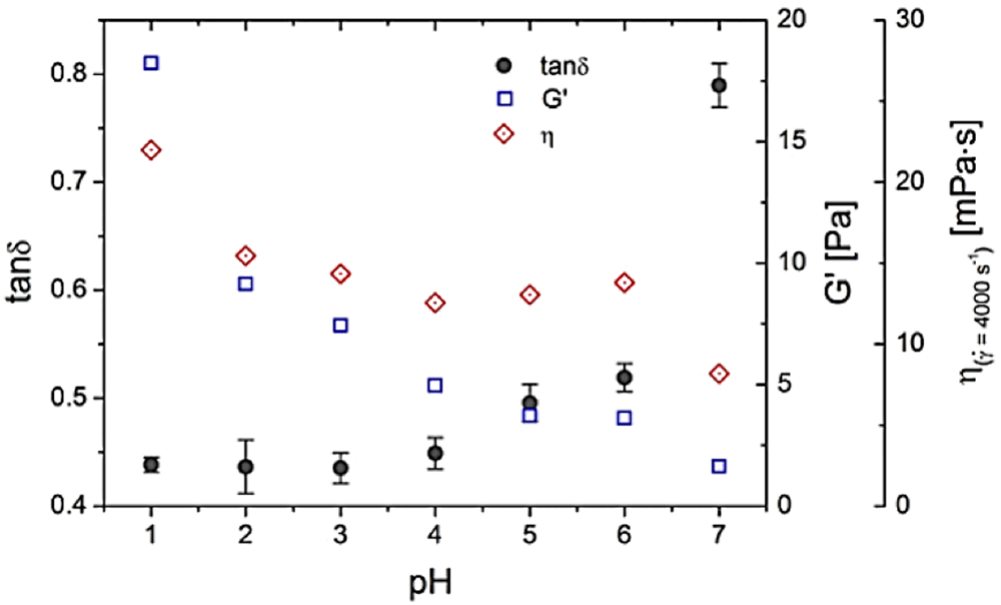
The effect of pH on rheological properties of ND-mucin solutions. The loss tangent $(\tan \,\delta =G^{\prime\prime} /G^{\prime} )$, storage modulus (G′), and viscosity at $\dot{\gamma }=400$ s^−1^ of 30 mg/mL ND-mucin solutions in 10 mM buffer at different pH values (pH 1–7).

Figure 7 illustrates the effect of reducing pH from 7 to 1 for 30 mg/mL ND-mucin solution. For all values of pH tested, we see similar shear thinning behavior, but distinct values of complex viscosity, $|{\eta }^{\ast }|$, and G′ (Supplementary Figures S10A and S10B). Contrary to expectations^42^, we observe no significant change in the values of viscoelasticity parameter $(\tan \,\delta )$ between pH 1 and 6. A significant decrease in $\tan \,\delta$ is observed between pH 7 and 6, which also coincides with a measurable increase in high shear viscosity. Below pH 6, the decrease in pH renders mucin solutions more viscous whereby G′ and G″ both increase by the same factor, with their ratio remaining roughly constant. The increase in G′ and G″ increases the yield strength of the network with the yield stress parameter increasing from 3 Pa to 6.4 Pa. Thus, acidification strengthens the macroscopic Muc2 mucus network, without substantial increase in the viscoelasticity of mucus solutions.

## Discussion: Model of mucin assembly and perspectives on the formation of controlled mucus-like systems

**Figure 8:**
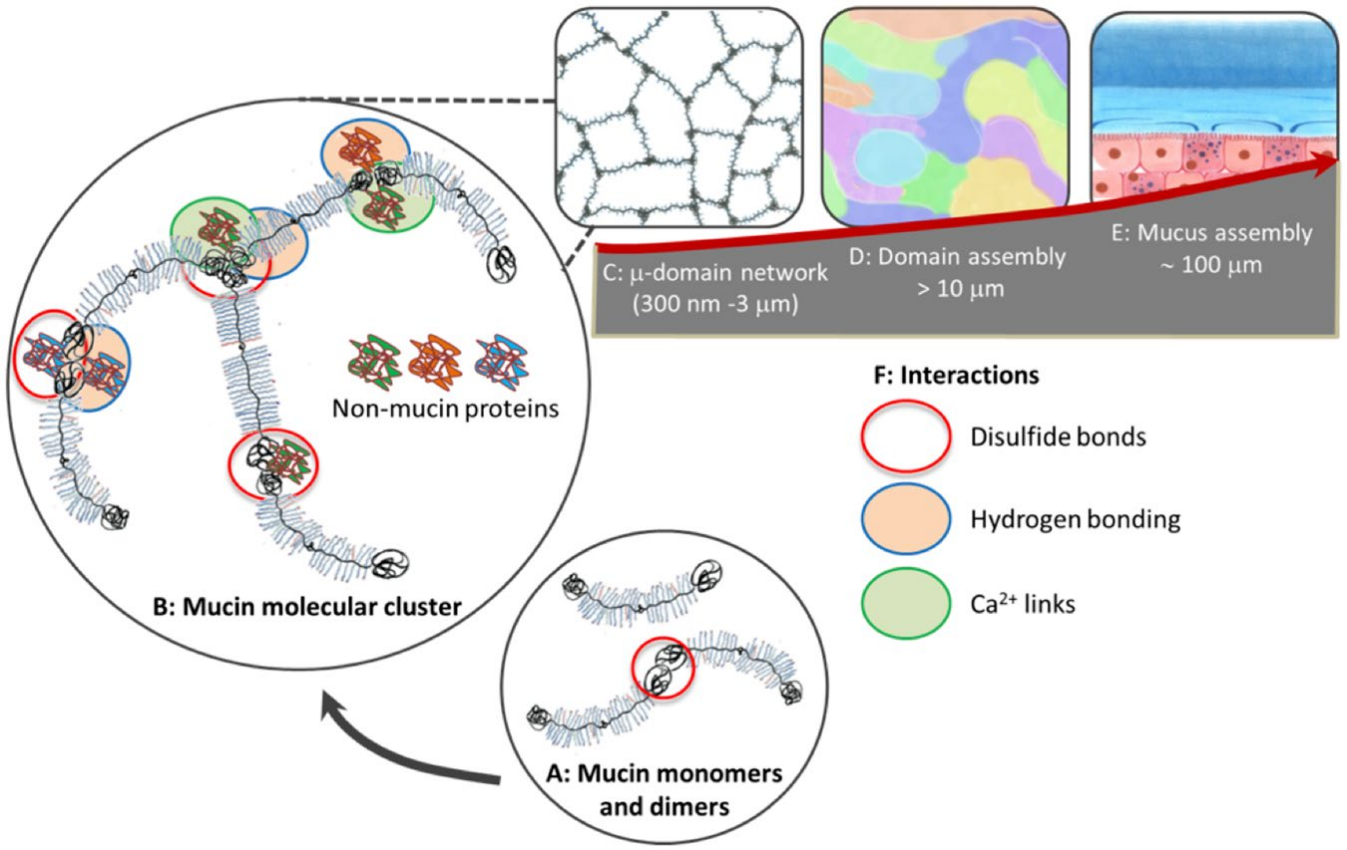
Hierarchical assembly of intestinal mucin. Mucin molecules and oligomers (**A**) assemble into molecular clusters (**B**) which form a micro-domain network (**C**) that assembles into a yield stress fluid (**D**) that exhibits mucus-like rheological behavior (**E**). A proposed structure of mucin molecular cluster oligomer (**B**) formed through interactions between mucin and non-mucin proteins (colored globules). The interactions (**F**) are governed by disulfide bonds and Ca^2+^-mediated links, with hydrogen bonding playing a crucial supporting role.

Multiscale rheological characterization of non-denatured Muc2 intestinal mucin preparations across a broad spectrum of conditions brings new insights into molecular interactions that drive mucin assembly, which is schematically conceptualized in Fig. 8. Firstly, we establish the hierarchical nature of this assembly that unfolds on different length scales, conferring it a unique set of mucus-like rheological properties at physiological concentrations (∼ 2 wt%). On the molecular scale our results agree well with previous findings^25^, which established that mucin oligomers are formed through disulfide bridges and, to a lesser extent, hydrogen bonds (Fig. 8A). On the micrometer scale, we observe viscoelastic micro-domains (Fig. 8C) that assemble into a yield stress fluid (Fig. 8D). Thus, the important distinction of the mucus system is the ‘duality’ of its rheological behavior whereby some properties, i.e. viscoelasticity on the micrometer scale, are characteristic of polymers, while other properties, such as yield stress, are typical for colloidal glasses and concentrated suspensions. The microstructural ‘swirl’ model (Fig. 8D) reconciles the co-existence of both types of behavior, which are found both in ND-mucin preparation as well as crude *ex vivo* mucus (Fig. 8E).

Further, we have uncovered the key factors of assembly of the micro-domains, which are Ca^2+^ links enabled by non-mucin components and facilitated by hydrogen bonding as schematically illustrated in Fig. 8B. We show that all interactions act in concert, whereby disruption of any of the individual elements results in the loss of network and loss (or significant reduction) of viscoelastic properties. Importantly, the unique mucus-like properties emerge only when mucin is isolated under native conditions, in which it remains associated with non-mucin secreted proteins that can only be removed using denaturing or chaotropic agents.

**Figure 9:**
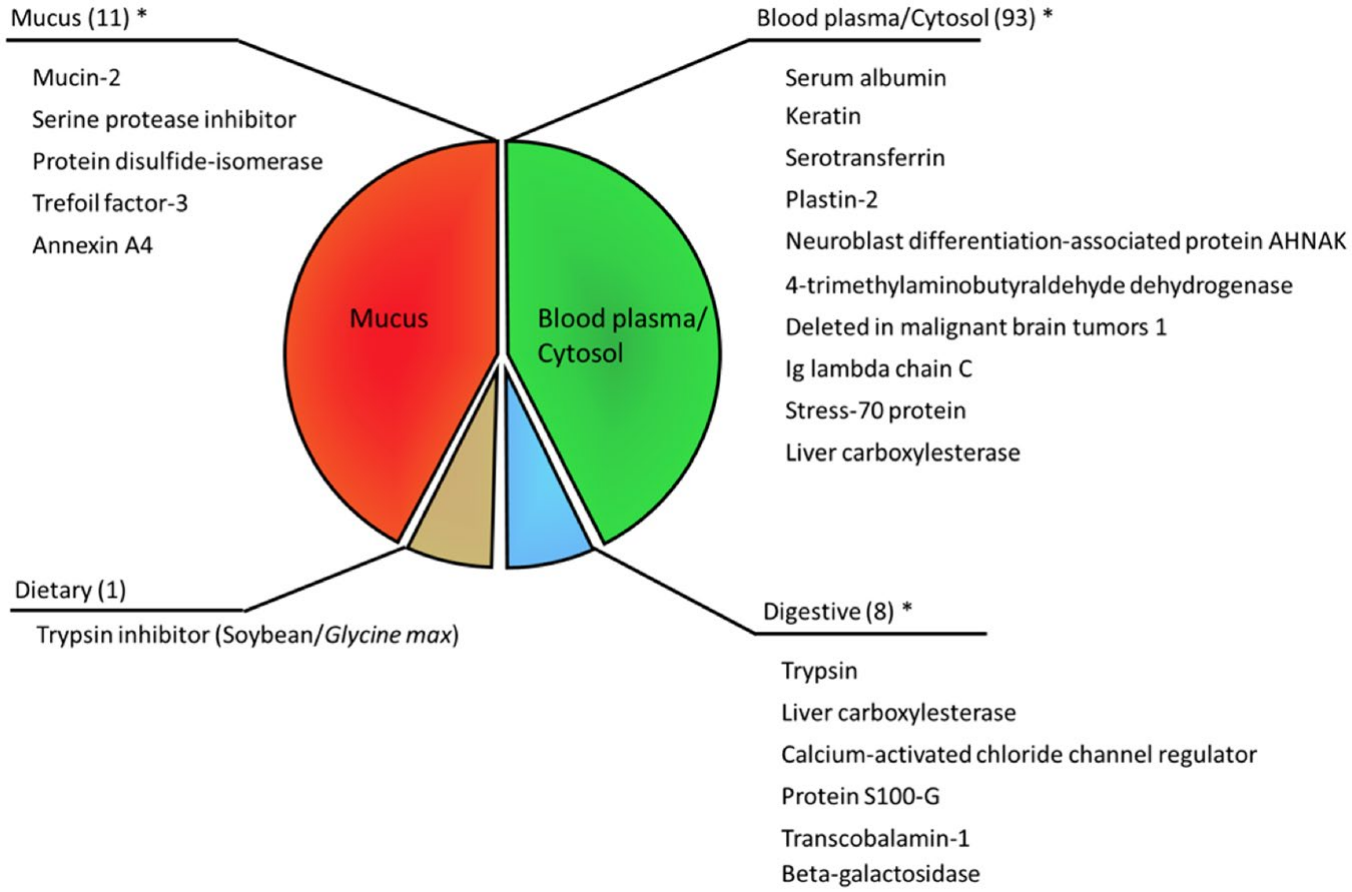
Pie chart of proteins identified in ND-mucin and grouped according to their predominant areas of expression and/or origin. Most abundant proteins are listed in the figure, with numbers in brackets corresponding to the total number of hits in each group. For protein groups marked with asterisk (*), a full list of candidate proteins is provided in Table S1 (See ESI). Mucus components are designated based on their possible involvement in the formation of mucus granules within the secretory cells and/or their involvement in granule transport (to the membrane) and mucus extrusion process. Blood plasma and cell cytosol components are a broad group of proteins that originate from cytosol components of lysed/sloughed-off epithelial cells as well as blood transudate/blood contamination from the surgical procedure. A small number of identifications are related to digestive enzymes, and proteins involved in nutrient uptake, as well as proteins originating from animal diet (soybean).

Based on tandem mass spectroscopy data and subsequent identification of protein components in the ND-mucin preparation, we have selected 19 proteins representing the most abundant species within the broader population of ca. 100 unique hits (a full table of identifications is given in Supplementary Table S1. Figure 9 shows a designation of these proteins based on their possible origin divided into 4 categories: (i) components of mucus or mucus vesicle, (ii) cytosol and blood components, as well as digestive (iii) and dietary components (iv). From this list we identify several components that may be implicated in mucus assembly and/or affect the molecular network after secretion, including: calcium binding protein, trefoil factor 3 (TFF3), and multiple type I and II keratins and a keratin-binding protein (plastin-2), a variety of enzymes (including protein disulfide isomerases) potentially involved in formation or destruction of covalent bonds. We also note the possibility of serum albumin and serpin to contribute to the mucus network as both are known to bind via hydrogen bonding to mucin and mucin-like glycoproteins.

The crucial role of Ca^2+^ puts new emphasis on calcium binding proteins, which can be a key candidate responsible for the formation of Ca^2+^-mediated links. The presence of TFF3 is also remarkable; the trefoil factors have long been implicated in mucus assembly due to their co-expression with mucus-forming glycoproteins in secretory cells across a range of animals, and therefore the overrepresentation of TFF3 in the ND-mucin preparation indicates its strong affinity to mucin, potentially contributing to mucus structure and rheology. Finally, we expect that keratins may contribute to the viscoelastic network and formation of the yield stress fluid in a similar fashion to the formation of hagfish slime^43^, where it has been shown that the unique rheological properties of the slime stem from a composite effect of filamentous proteins (e.g., keratin) and polyelectrolyte properties of mucin. We also draw attention to the dietary trypsin inhibitor from soy which is likely to originate from animal feed. The significance of this finding is that dietary proteins may strongly associate with mucus, and hence contribute to the mucus functionality, and could therefore be used purposefully to alter mucus properties. Based on these identifications we propose to further scrutinize the role of Ca^2+^ binding protein and keratin in the mucus assembly, as well as continue efforts to elucidate the role of TFF3, and finally investigate dietary sources of proteins with functionality to bind mucus and/or modulate Ca^2+^ links.

## Materials and Methods

### Mucin purification and quantification

All animal procedures were approved by the Animal Ethics Committee of the University of Queensland (ANRFA/QAAFI/424/14). All experiments were performed in accordance with the approved procedures, relevant guidelines and regulations. Porcine intestinal mucin was selected as a model for human intestinal mucin. Fresh porcine small intestines were collected immediately after euthanasia with histology sections taken from the proximal small intestine (Supplementary Section 1). The intestines were cut into sections (ca. 60 cm long) and inverted to expose the internal mucus layer before briefly washed with PBS buffer and gently pulled between two smooth glass rods to remove the loose mucus layer with care taken to avoid removing epithelial tissue. Mucus was diluted in a 1:1 (v/v) ratio with either 6 M GuHCl solution (denatured preparation) or 0.2 M NaCl solution (native preparation) for 24 h at 4 °C. The NaCl solution contained 0.04% sodium azide, 0.1 M EDTA, 1 M aminohexanoic acid, 0.05 M benzamidine and a protease inhibitor cocktail. The dissolution step was followed by two rounds of CsCl isopycnic density-gradient centrifugation (1.4 g/mL starting density) in a Beckman L-100 ultracentrifuge (Beckman Ti45 rotor, 72 h, 40,000 rpm, 12 °C)^25^. Detailed fraction analysis is provided in Supplementary Section 2. Briefly, following the first round of centrifugation, 20 fractions of increasing density were analyzed for UV absorbance (280 nm), density, glycoprotein content using dot-blot PAS staining (Supplementary Figure S1A) and protein content using SDS-PAGE (Supplementary Figure S2A). Based on the dot-blot analysis, mucin rich fractions were pooled (typically fractions 8 to 15), and subjected to a 2^nd^ round of CsCl isopycnic density-gradient centrifugation (1.5 g/mL starting density) with 20 fractions of increasing density analyzed for UV absorbance (280 nm), density, glycoprotein content (Supplementary Figure S1B) and protein content using SDS-PAGE (Supplementary Figure S2B). The mucin-rich fractions were pooled (typically fractions 3 to 7), exhaustively dialyzed against deionized water, frozen at −80 °C and freeze-dried. Native and denatured mucin preparations were analyzed for their peptide identity according to Kesimer and Sheehan^44^ (See Supplementary Section 3).

Conventional methods of determining protein concentration such as UV absorbance, bicinchoninic acid or Bradford reagent assays underestimate mucin content due to the large proline, serine and threonine-rich glycosylation domains^45^. Thus, microtiter plate colorimetry was used to quantify mucin content of native and denatured mucin preparations using PAS staining and Bradford assay to measure the mucin and non-mucin protein component, respectively. Based on a modified quantification method by Kilcoyne *et al*.^46^, native and denatured mucin, and a mucin standard using denatured mucin purified using a 100 kDa MWCO diafiltration kit (Merck Millipore, Massachusetts, USA). 80 μg of each preparation was incubated with 100 μL of periodic acid solution (0.05% (w/w) periodic acid and 7% (w/w) acetic acid) and incubated at 37 °C for two h. 100 μL of Schiff reagent was added, shaken for 5 min before being allowed to develop for 25 min with absorbance measured at 550 nm. Protein content was quantified using a Bradford assay (Bio-Rad) using 100 μg of each mucin preparation was pipetted into the well and incubated with 150 μL of Coomassie Plus Reagent for 5 min and measured at 595 nm. Standard curves for glycoprotein and mucin quantification were assessed by a best-fitting regression analysis and correlation coefficients calculated using Graphpad Prism (version 7).

### Narrow-gap parallel plate rheometry and microrheology

Bulk rheometry was carried out using a stress-controlled rheometer with parallel plate narrow-gap technique^47–50^. Briefly, measurements were carried out at 25 °C using a stress-controlled rheometer (Haake MARS III, Thermo Fisher Scientific, Karlsruhe, Germany) with a parallel-plate (35 mm titanium) geometry at a gap of 40 μm. A temperature hood was used to maintain the surrounding environment with silicon oil (10 cSt) to prevent evaporation. The storage modulus, G′, and loss modulus, G″, were determined by performing small deformation frequency sweeps from 10 to 100 rad/s within the linear viscoelastic region as determined from the stress amplitude sweep of each sample. Purified lyophilized mucin preparations were dissolved in the desired buffer solution and allowed to equilibrate for at least 48 h (4 °C) prior to experimentation. Generally, mucin was suspended in 10 mM phosphate buffer at pH 7. In experiments where the effect of pH on mucin properties was evaluated, the desired pH was achieved by mixing appropriate volumes of 100 mM KCl/HCl and 100 mM acetic acid/sodium acetate solutions to obtain the desired pH with a final concentration of 10 mM buffer in solution, to maintain the same buffer composition and ionic strength. Analytical grade Ca^2+^, EDTA, DTT and GuHCl were prepared in 10 mM pH 7 phosphate buffer.

The microrheology of ND- and GH-mucin solutions were characterized using 0.5 μm uncoated carboxyl-functionalized polystyrene microspheres (1:2500 dilution from 2.6% stock, Polysciences Inc., Warminster, Pennsylvania, USA) embedded in 10 μL of mucin solution using plane non-concave microscope slides with secure-seal spacers (ThermoFisher Scientific) between the slide and coverslip. The carboxyl-functionalized particles were chosen over amine surface coated parties due to previous studies showing the amino-coated particles have impaired diffusion^51^ and exhibit similar behavior to their uncoated counterparts^5^. A Nikon Eclipse Ti inverted fluorescence microscope equipped with a 100× oil immersion objective was used with a CoolLED light source (Lumencor). The Brownian motion was recorded using a Phantom v7.3 fast-action camera at 100 frames per second while avoiding edge effects. The *x*- and *y*- coordinates of individual particle trajectories were obtained using PolyParticleTracker software^52^ for MATLAB (version 2016a) routine that used a modified version of the algorithm developed by Crocker and Grier^53^. For stochastic motion of a probe in a Newtonian fluid, the ensemble averaged mean square displacement (MSD) as a function of lag time, ${\rm{\Delta }}{r}^{2}(\tau )$, is linearly dependent on the lag time at each time interval, ${\rm{\Delta }}t$, and diffusion coefficient, *D*, in a two-dimensional system is described by:

2 $$\langle \Delta {r}^{2}(\tau )\rangle =4D\tau $$

For viscoelastic fluids, the power law time dependence of the MSDs is observed where α is the exponent:

3 $$\langle \Delta {r}^{2}(\tau )\rangle =M{\tau }^{\alpha }$$

$\alpha$ = 1 describes colloidal particle diffusion in a purely viscous fluid. For viscous fluids, the diffusion coefficient $\,(D$) of a probe can then be used to determine the fluid viscosity using the Stokes-Einstein equation:

4 $$\eta =\frac{{k}_{B}T}{6\pi D{r}_{h}}$$

where $\,\eta$ is dynamic viscosity of the fluid medium, ${r}_{h}$ is the hydrodynamic radius of the tracer particle, $T$ is absolute temperature, and ${k}_{B}$ is the Boltzmann constant. For viscoelastic materials, $0 < \alpha  < 1$, the generalised Stokes-Einstein relation (GSER) can be applied to account for time dependency of diffusivity (*M*) by evaluating the time-dependent compliance, $J(\tau )$, of the material, which can be readily extracted from the experimental MSD spectra^54^:

5 $$J(\tau )=\frac{\pi {r}_{h}}{{k}_{b}T}\langle {\rm{\Delta }}{r}^{2}(\tau )\rangle $$

Further information on data analysis is provided in Supplementary Sections 4, 5 and 6.

## Electronic supplementary material

Supplementary Information

Table_S1

Table_S2
