# Supplementary material for: Mucin gel assembly is controlled by a collective action of non-mucin proteins, disulfide bridges, Ca2+-mediated links, and hydrogen bonding
Source: Sci Rep. 2018 Apr 11;8:5802. doi: 10.1038/s41598-018-24223-3 (PMC5895598; doi:10.1038/s41598-018-24223-3)
Supplement: Supplementary file 1 — Supplementary Information [file 41598_2018_24223_MOESM1_ESM.docx]

**Supplementary Information**

**Mucin gel assembly is controlled by a collective action of non-mucin proteins, disulphide bridges, Ca^2+^-mediated links, and hydrogen bonding**

Oliver W. Meldrum ^a,b^, Gleb E. Yakubov ^a,c,1^, Mauricio R. Bonilla ^a,c^, Omkar Deshmukh ^c^, Michael A. McGuckin ^d^, Michael J. Gidley ^a,b^

^a^ ARC Centre of Excellence in Plant Cell Walls, The University of Queensland, St Lucia, 4072, Qld, Australia;

^b^ Centre for Nutrition and Food Sciences, Queensland Alliance for Agriculture and Food Innovation, The University of Queensland, St Lucia, 4072, Qld, Australia;

^c^ School of Chemical Engineering, The University of Queensland, St Lucia, 4072, Qld, Australia;

^d^ Chronic Disease Biology and Care Program, Mater Research Institute - The University of Queensland, Translational Research Institute, Woolloongabba, QLD 4102, Australia.

^1^ Corresponding Author: [gleb.yakubov@uq.edu.au](mailto:gleb.yakubov@uq.edu.au), ARC Centre of Excellence in Plant Cell Walls, School of Chemical Engineering (EAIT), The University of Queensland, Brisbane 4072, Australia, +61 7 3365 4920

**Supplementary Section 1: Reagents and histology**

All reagents were purchased from Sigma-Aldrich (St Louis, Missouri, USA) and used as received unless otherwise stated. Solutions were prepared using deionized and filtered water (filter pore size: 0.22 μm, resistivity of 18.2 MΩs⋅cm, Sartorius Stedim Biotechnology). Following the dissection and excision of the small intestine, segments of apical small intestinal sections were immediately fixed in methanol-Carnoy’s fixative (60:30:10 methanol: chloroform: glacial acetic acid) to preserve the surface mucus layer prior to paraffin-embedding. For histology, tissue samples were sectioned, dewaxed and mounted onto microscope slides before staining with Alcian blue/PAS stain according to a well-established protocol (Matsuo, Ota, Akamatsu, Sugiyama, & Katsuyama, 1997). Bright field images were taken using an upright AxioScope A1 microscope (Zeiss, Germany).

**Supplementary Section 2: Fractions analysis**

Following centrifugation, 20 fractions of increasing density were analysed for UV absorbance (280 nm), density, protein and glycoprotein content using a combination of dot-blot analysis and SDS-PAGE (Supplementary Figure S1). Dot-blot analysis and agarose gel electrophoresis were used to identify the presence of mucin immobilised onto nitrocellulose paper and stained using Periodic acid-Schiff (PAS) staining. Agarose gel electrophoresis was performed in a Bio-Rad Subcell gel tank with 0.7% (w/v) agarose gel in TAE-SDS buffer (20 mM Tris-Base, 1 mM EDTA, 0.1% SDS, pH 8) at 50 V for 4-8 h. After separation, the gel was first reduced using 10 mM DTT for 20 min and incubated for 5 min in transfer buffer (0.6 M NaCl, 50 mM sodium citrate, pH 8) before being transferred to a nitrocellulose membrane (Bio-Rad) using the Bio-Rad vacuum blotter (Model 7850) connected to a vacuum pump (10 mbar) for 4 h (Davies, Wickstrom, & Thornton, 2012). Each sample was loaded in equal volumes to allow for a comparison of the staining intensities between samples. For dot-blot analysis, 40 μL aliquot of fractions were vacuum transferred onto pre-wet 0.45 μm nitrocellulose paper (20 mbar). Once absorbed, the membrane was washed in dH_2_O (deionised water, 18.2 MΩs⋅cm) for two min each and transferred to a freshly prepared solution containing 1% (w/w) periodic acid and 3% (w/w) acetic acid for 20 min at room temperature. The membrane was then rinsed twice in freshly prepared 0.1% (w/v) sodium metabisulfite in 1 mM HCl for 2 min each. Schiff reagent was then added for 15 min, rinsed twice again in sodium metabisulfite/HCl solution before being air-dried and imaged. The identification and distribution of proteins were analysed using 4-12% (w/v) NuPAGE Bolt Bis-Tris precast gels (Invitrogen) with NuPAGE SDS Sample Buffer. Unless otherwise stated, all samples were reduced with NuPAGE Sample Reducing Agent at 70°C for 5 min prior to loading with 100 V applied for 95 min. Gels were then stained with Coomassie Plus (Invitrogen) and imaged using a Bio-Rad ChemiDoc MP Imaging System (Supplementary Figure S2). Proteins from SDS-PAGE gels were then transferred to nitrocellulose membranes using the iBlot 2 Dry Blotting System at 20 V for 15 min (Thermo Fisher Scientific). Agarose gel electrophoresis was performed in a Bio-Rad Subcell gel tank with 0.7% (w/v) agarose gel in TAE-SDS buffer (20mM Tris-Base, 1mM EDTA, 0.1% SDS, pH 8) at 50V for 4-8 h. After separation, the gel was first reduced using 10 mM DTT for 20 min and incubated for 5 min in transfer buffer (0.6 M NaCl, 50 mM sodium citrate, pH 8) before being transferred to a nitrocellulose membrane (Bio-Rad) using the Bio-Rad vacuum blotter (Model 7850) connected to a vacuum pump (10 mbar) for 4 h (Davies et al., 2012). Each sample was loaded in equal volumes to allow for a comparison of the staining intensities between samples. Mucin-rich fractions identified by PAS staining were pooled and density was adjusted using CsCl (1.5 g/mL) before being centrifuged again for 68-72 h at 40,000 rpm (12 °C).

**Supplementary Section 3: Trypsin digestion of proteins and LC-MS/MS analysis**

ND- (native) and GH-mucin (denatured) preparations were analysed for their peptide identity. Freeze-dried mucin preparations were first dissolved in reducing buffer (6M GuHCl, 0.1M Tris-HCl, 5 mM EDTA, pH 8) for 6 h at room temperature before being dialysed against dH_2_O. Mucin preparations were then reduced and alkylated in 10 mM dithiothreitol (DTT) for 2 h at 37 °C followed by incubation in 20 mM iodoacetamide (IAA) for 30 min in the dark at room temperature. The solution was then dialysed into 50 mM tris-HCl to remove excessive salts before incubation with trypsin (1 μg/mL) overnight at 37°C. The trypsin digested peptides were subsequently isolated and concentrated from contaminating glycopeptides and salt using a C18 resin embedded in a Zip-Tip pipette (Merck Millipore) before final dilution into 1:10 formic acid solution (1% w/w). The eluted peptides were directly analysed on a TripleTof 5600 instrument (ABSciex) using a Nanospray III interface. The gas and voltage settings were adjusted as required. The MS TOF scan across m/z 350-1800 was performed for 0.5 seconds followed by information dependent acquisition for the top 20 peptides for 0.5 peptides across m/z 40-1800 (0.05 seconds per spectra). The data output was converted to .mgf format and searched using MASCOT accessed via the Australian Proteomics Computational Facility. Data was searched in the SwissProt database, searching all species with trypsin, 2 mis-cleavages, MS tolerance of 50 ppm, MS/MS tolerance of 0.1 Da with oxidation (met, variable) and carbamidomethylation (cys, fixed) modifications included (Supplementary Tables 1 and 2).

**Supplementary Section 4: Rotational rheometry and microrheology**

**Rotational rheometry**

The Cox-Merz rule was applied to correlate the SAOS data with that obtained from steady shear measurements by overlaying the steady shear and SAOS viscosities against the shear rate ($\dot{\gamma}$) or angular oscillation frequency (ω). The Herschel-Bulkley model was applied to ND-mucin to quantify the amount of stress that the fluid may experience before it yields and begins to flow. The constitutive equation of the Herschel-Bulkley model is written as:

| $\sigma= \sigma_{0}+ {K_{\mathrm{HB}}\gamma}^{n}$ | (Eq S1) |
| --- | --- |
|  |  |

where $\sigma$ is the shear stress, $\gamma$, the shear rate, $\sigma_{0}$, the yield stress, $k$, the consistency index and $n$ the flow index. For $n<1$, the fluid is shear thinning.

**Particle tracking microrheology**

The magnitude of the mechanical modulus, $\tilde{G}\left( \omega\equiv\frac{1}{\tau} \right)$, can be derived in a following form:

| $\left\vert\tilde{G}\left( \omega\right) \right\vert=\frac{k_{B}T}{\pi r_{h}\left\langle\tilde{r}^{2}\left( 1/\omega\right) \right\rangle\Gamma\left( 1+\alpha\left( \omega\right) \right)}$ | (Eq S2) |
| --- | --- |

where $\Gamma$() the gamma function and $\tilde{∎}$ denotes the unilateral Fourier Transform for the complex frequency, $\omega$. The frequency-dependent loss, G”, and storage, G’, moduli and complex viscosity can be calculated algebraically using:

| $G^{'}\left( \omega\right)=\left\vert\tilde{G}\left( \omega\right) \right\vert cos\left( \frac{\pi\alpha\left( \omega\right)}{2} \right)$ | (Eq S3) |
| --- | --- |
| $G^{''}\left( \omega\right)=\left\vert\tilde{G}\left( \omega\right) \right\vert sin\left( \frac{\pi\alpha\left( \omega\right)}{2} \right)$ | (Eq S4) |
| $\eta^{*}=\frac{\sqrt{{G'\left( \omega\right)}^{2}+{G''\left( \omega\right)}^{2}}}{\omega}$ | (Eq S5) |

The value of frequency $\omega^{*}=\frac{1}{t^{*}}=\left( \left( 1+\alpha\right) \right)^{\frac{1}{\alpha-1}}$ (for α = 0.5 *ω*^*^~ 1.27 Hz and *t*^*^ ~ 0.7854 s). At the frequency *ω*^*^, the form of the expression for ${}^{*}$coincides with the classic Stokes-Einstein equation (Eq 4), enabling consistent comparison between for *c* ≤ 10 mg/mL and ${}^{*}$ for *c* ≥ 15 mg/mL. For MSD spectra where α ~ 1, the diffusivity is inversely proportional to the solution viscosity via the Stokes-Einstein equation (Eq 4). For concentrations with sub-diffusive particle motion (α < 1), the viscosity depends on frequency, for α ~ 0.5 G’(ω) ≈ G”(ω), which enables us to define the effective viscosity using the expression for ${}^{*}$ as ${}_{\mathrm{eff}}^{*}={}^{*}\left( \omega^{*}\approx1.27 \mathrm{Hz} \right)$ (Eq S3, S4 and S5).

**Supplementary Section 5: Analysis of the rheological behavior of dilute ND-mucin solutions**

The Fuoss scaling is characteristic for semi-dilute solutions of flexible linear polyelectrolytes and comb-polyelectrolytes with short side-chains. In our experiments, the linear scaling ${}_{\mathrm{sp}}\sim c$ found in low concentration ND-mucin suggests that solutions show ‘dilute’ behavior. Notably, the absolute values of viscosity for *c* ≤ 10 mg/mL are very similar to those reported by Georgiades et al. (Georgiades, Pudney, Thornton, & Waigh, 2014) for purified duodenal mucin (Verdugo, Aitken, Langley, & Villalon, 1987), suggesting that the partial polymer phase volume of both preparations is similar $\left( \sim\left[ \right]c \right)$. Non-mucin proteins are relatively minor components of ND-mucin preparation, ca. 7 wt% according to quantification presented in Table 1. Therefore, on their own - as a “solution” of globular proteins - they contribute only a small correction to the partial phase volume of mucin and hence cannot be responsible for the observed rheological behavior. Further analysis was undertaken by examining the reduced $\left( {}_{r} \right)$ and intrinsic viscosity $\left( \left[ \right] \right)$of ND-mucin; the latter is a fundamental physical parameter that characterizes the volume of a polymer chain in solution and is directly proportional to the hydrodynamic radius and shape of the molecule.

| $\left[ \right]=\lim_{\begin{aligned} c \to0 \\ \omega\to0 \end{aligned}} {}_{r}=\lim_{\begin{aligned} c \to0 \\ \omega\to0 \end{aligned}} \frac{{}_{\mathrm{sp}}}{c}$ | (Eq S6) |
| --- | --- |

Since specific viscosity scales are linear with concentration, the reduced viscosity is constant. In the range of concentration $1\leq c\leq10$ mg/mL we find $\left[ \right]$= 5.7 dL/g. Taking this number as a guide and assuming that molecular weight of mucins is ca. 1 MDa. This estimation also assumes that Eq S6 is applicable for mucin as well as for the experimental conditions of particle tracking technique, we estimate the hydrodynamic radius of mucin using the Flory-Fox equation

| $R_{H}=\sqrt[3]{\left[ \right]M_{w}{}^{-1}}$ | (Eq S7) |
| --- | --- |

Φ ≡ 2.9⋅10^24^ is the Flory parameter and *M*_w_ is molecular weight. The estimated *R*_H_ = 60 nm is in broad agreement with previous data (Di Cola, Yakubov, & Waigh, 2008; Pantelis Georgiades et al., 2014), and is consistent with the presence of mucin oligomers.

The presence of oligomers is further inferred from SDS-PAGE analysis (Figure 1), which shows the reduction in the effective molecular weight of the mucin band upon treatment with disulfide bond reducing agent, DTT (Ambort et al., 2011). The large number of non-mucin proteins released upon DTT treatment indicates non-mucin proteins are either covalently linked to mucin via disulfide bonds or their binding is dependent on the entanglement of mucin formed by inter- or intra-molecular disulfide bonds. Previous work has established mucin is capable of independent higher order assembly, here we show that non-mucin components are instrumental for stabilising physical crosslinks between mucin domains, and potentially responsible for promoting formation of additional links (Ridley et al., 2014; Schutte et al., 2014). Rheologically, these complex structures behave as “particles”, and hence result in linear scaling of ${}_{\mathrm{sp}}$ with concentration. Upon increase in the concentration the mucin oligomers form a network between 10 and 15 mg/mL, i.e., at the point where tracer particle mobility decreases due to viscoelastic effects (α ~ 0.5). For *c* ≥ 15 mg/mL, the linear scaling of the specific viscosity with concentration is consistent with molecular assemblies of dense viscoelastic gels (Rubinstein, Colby, Dobrynin, & Joanny, 1996).

**Supplementary Section 6: Analysis and quantification of the microrheological heterogeneity of ND-mucin solutions**

In structurally homogenous materials, where all particles are in the same micro-environment, the van Hove distribution is Gaussian (solid and dash lines), while in complex materials the deviation from a Gaussian distribution indicates the presence of heterogeneities; i.e., different tracer particles probe different regions of the fluid with distinct rheological properties. A useful parameter to characterize the degree of deviation is excess kurtosis$, I_{2}\left( \tau\right)$, which is a measure of the ‘tail’ of the probability distribution (Kegel & van Blaaderen, 2000); for the Gaussian distribution$I_{2}\left( \tau\right)=0$.

| $I_{2}\left( \tau\right)=\frac{\left\langle{\Delta x}^{4}\left( \tau\right) \right\rangle}{3\left\langle{\Delta x}^{2}\left( \tau\right) \right\rangle^{2}}-1$ | (Eq S8) |
| --- | --- |

To probe the effect of the cage boundary on the tracer particle diffusion, two successive displacements of equal lag time are correlated. If the diffusion is caged, then the increase in the displacement $\boldsymbol{r}_{01}\left( \tau\right)$at the time $t_{1}=t_{0}+\tau;$

| $\left\vert\boldsymbol{r}_{01}\left( \tau\right) \right\vert=\sqrt{{\Delta x}_{01}^{2}\left( \tau\right)+{\Delta y}_{01}^{2}\left( \tau\right)}$ | (Eq S9) |
| --- | --- |

results in a higher chance of particle bouncing back during the successive time interval, $t_{2}=t_{1}+\tau$, which increases the chance of the value $\left\langle{\tilde{\boldsymbol{r}}}_{12}^{x} \right\rangle$ to be negative and scale linearly with $\boldsymbol{r}_{01}$:

| $\left\langle{\tilde{\boldsymbol{r}}}_{12}^{x} \right\rangle=\left\langle\frac{\boldsymbol{r}_{01}\boldsymbol{r}_{12}}{\left\vert\boldsymbol{r}_{01} \right\vert} \right\rangle=-\beta\boldsymbol{r}_{01}$ | (Eq S10) |
| --- | --- |

where *β* is a positive constant. If *β* is zero then the particle motion is random, and hence non-caged.

**Table S1.** Identification of protein components in ND-mucin using tandem LC-MS/MS. Secretion locations are denoted as following: *B* – cytosol blood; *M* – mucus; *D* – dietary components; *I* – intestinal/digestive enzymes.

See Table S1 excel file

**Table S2.** Identification of protein components in GH-mucin using tandem LC-MS/MS. Secretion locations are denoted as following: *B* – cytosol blood; *M* – mucus; *D* – dietary components; *I* – intestinal/digestive enzymes.

See Table S2 excel file

**Table S3.** Steady shear viscosity at $\dot{\gamma}=4000 s^{-1}$ of 10 mg/mL – 50 mg/mL ND- and GH-mucin.

| \| Concentration  mg/mL \| $\left[ \right]_{\dot{\gamma} = 4000 s^{-1}}^{*}$ [dL/g] \| \| Ratio \| \| --- \| --- \| --- \| --- \| \| ND-mucin \| GH-mucin \| \| 50 \| 2.0 \| 2.6 \| 1.3 \| \| 40 \| 1.1 \| 1.8 \| 1.7 \| \| 30 \| 0.6 \| 1.7 \| 2.9 \| \| 20 \| 0.7 \| 1.4 \| 2.1 \| \| 10 \| 0.7 \| 1.6 \| 2.3 \| |
| --- | --- | --- | --- | --- | --- | --- | --- | --- | --- | --- | --- | --- | --- | --- | --- | --- | --- | --- | --- | --- | --- | --- | --- | --- | --- | --- |

**Figure S1**

| **A** |  |  |  |
| --- | --- | --- | --- |
| **B** |  |  |  |
| **Figure S1.** Representative fraction analysis obtained from a CsCl isopycnic density-gradient centrifugation of small intestinal mucus extracted in 50:50 ratio extraction buffer containing a protease inhibitor cocktail. Following the 1^st^ (A, starting density 1.4 g/mL) and 2^nd^ (B, starting density 1.5 g/mL) rounds of centrifugation, samples were fractionated by increasing density (green line and triangle) and analysed for A280 UV absorbance (Blue line and circle) and PAS intensity (red line and square) of the Dot blot nitrocellulose paper was used to identify glycoprotein rich fractions. In this case, fractions 5-16 were pooled from the 1^st^ round and fractions 3-7 pooled from the 2^nd^ round. | | | |

**Figure S2**

| **A** |  |  |  |
| --- | --- | --- | --- |
| **B** |  |  |  |
| **Figure S2.** Representative protein content of each fraction was analysed using SDS-PAGE from the 1^st^ (A, starting density 1.4 g/mL) and 2^nd^ (B, starting density 1.5 g/mL) round of CsCl isopycnic density-gradient centrifugation. 10 μL of each fraction were reduced (DTT) and subjected to electrophoresis with the gels stained with Coomassie blue. A typical output of the 1^st^ round of CsCl centrifugation shows the distribution of globular proteins are concentrated in fractions 1-5 and 18-20. After pooling mucin rich fractions from the 1^st^ round (Fraction 5-16), the 2^nd^ round of CsCl centrifugation highlighted the successful removal of the majority of detectable non-mucin proteins from the preparations with fractions 3-7 pooled. Small peptides observed near the dye front in fractions 1-9 were not successfully removed with this technique and may represent cleaved peptides from the N- and C-terminal regions of the mucin retained by disulphide linkages. (M) Molecular weight standards (kDa). | | | |

**Figure S3**

|  | 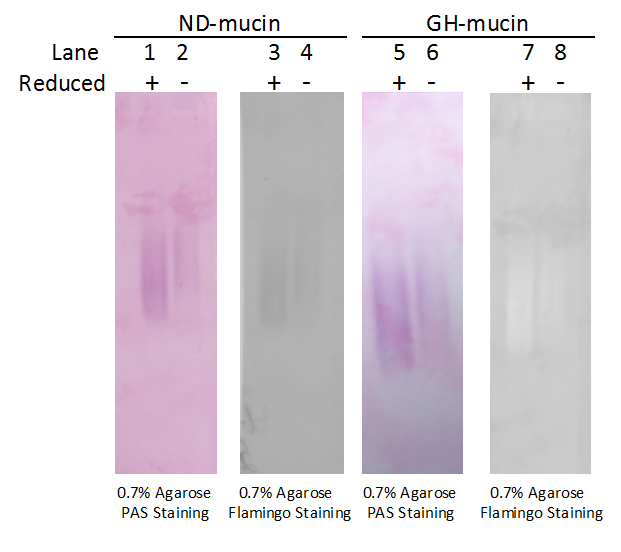 |  |  |
| --- | --- | --- | --- |
| **Figure S3.** Agarose gel electrophoresis of porcine intestinal mucin from the native (ND-) and denatured (GH-) mucin preparations isolated after the 2^nd^ round of CsCl Isopycnic density gradient centrifugation. Electrophoresis was carried out using 0.7% agarose gel in TAE buffer and 0.1% SDS, transferred onto nitrocellulose with PAS stained to detect carbohydrates and Flamingo staining for general proteins. ND- and GH-preparations were further compared with the inclusion of a reducing agent; 100 μg of lyophilised mucin preparations were loaded with equal volumes in each lane. Lanes: 1 and 3, reduced ND-mucin preparation, 2 and 4, non-reduced ND-preparation, 5 and 7, reduced GH-mucin preparation, 6 and 8, non-reduced GH-mucin preparation. | | | |

**Figure S4**

| MGLPLARLAAVCLALSLAGGSELQTEGRTRYHGRNVCSTWGNFHYKTFDGDVFR**FPGLCDYNFASDCRGSYKEFAVHLK**RGPGQAEAPAGVESILLTIKDDTIYLTRHLAVLNGAVVSTPHYSPGLLIEKSDAYTKVYSRAGLTLMWNREDALMLELDTKFRNHTCGLCGDYNGLQSYSEFLSDGVLFSPLEFGNMQKINQPDVVCEDPEEEVAPASCSEHRAECERLLTAEAFADCQDLVPLEPYLRACQQDRCRCPGGDTCVCSTVAEFSRQCSHAGGRPGNWRTATLCPKTCPGNLVYLESGSPCMDTCSHLEVSSLCEEHRMDGCFCPEGTVYDDIGDSGCVPVSQCHCRLHGHLYTPGQEITNDCEQCVCNAGRWVCKDLPCPGTCALEGGSHITTFDGKTYTFHGDCYYVLAKGDHNDSYALLGELAPCGSTDKQTCLK**TVVLLADKK**KNAVVFKSDGSVLLNQLQVNLPHVTASFSVFRPSSYHIMVSMAIGVRLQVQLAPVMQLFVTLDQASQGQ**VQGLCGNFNGLEGDDFK**TASGLVEATGAGFANTWKAQSTCHDKLDWLDDPCSLNIESANY**AEHWCSLLKK**TETPFGR**CHSAVDPAEYYKR**CKYDTCNCQNNEDCLCAALSSYARACTAKGVMLWGWREHVCNKDVGSCPNSQVFLYNLTTCQQTCRSLSEADSHCLEGFAPVDGCGCPDHTFLDEKGRCVPLAKCSCYHR**GLYLEAGDVVVR**QEERCVCRDGRLHCRQIRLIGQSCTAPKIHMDCSNLTALATSKPRALSCQTLAAGYYHTECVSGCVCPDGLMDDGRGGCVVEKECPCVHNNDLYSSGAKIKVDCNTCTCKRGRWVCTQAVCHGTCSIY**GSGHYITFDGKYYDFDGHCSYVAVQDYCGQN**SSLGSF**SIITENVPCGTTGVTCSK**AIKIFMGRTELKLEDKHRVVIQRDEGHHVAYTTREVGQYLVVESSTGIIVIWDKRTTVFIKLAPSYKGTVCGLCGNFDHRSNNDFTTR**DHMVVSSELDFGNSWK**EAPTCPDVSTNPEPCSLNPHRRSWAEKQCSILKSSVFSICHSKVDPKPFYEACVHDSCSCDTGGDCECF**CSAVASYAQECTKEGACVFWR**TPDLCPIFCDYYNPPHECEWHYEPCGNRSFETCRTINGIHSNISVSYLEGCYPRCPKDRPIYEEDLKKCVTADKCGCYVEDTHYPPGASVPTEETCKSCVCTNSSQVVCRPEEGKILNQTQDGAFCYWEICGPNGTVEKHFNICSITTRPSTLTTFTTITLPTTPTSFTTTTTTTTPTSSTVLSTTPKLCCLWSDWINEDHPSSGSDDGDREPFDGVCGAPEDIECRSVKDPHLSLEQHGQKVQCDVSVGFICK**NEDQFGNGPFGLCYDYKIR**VNCCWPMDKCITTPSPPTTTPSPPPTTTTTLPPTTTPSPPTTTTTTPPPTTTPSPPITTTTTPLPTTTPSPPISTTTTPPPTTTPSPPTTTPSPPTTTPSPPTTTTTTPPPTTTPSPPMTTPITPPASTTTLPPTTTPSPPTTTTTTPPPTTTPSPPTTTPITPPTSTTTLPPTTTPSPPPTTTTTPPPTTTPSPPTTTTPSPPTITTTTPPPTTTPSPPTTTTTTPPPTTTPSPPTTTPITPPTSTTTLPPTTTPSPPPTTTTTPPPTTTPSPPTTTTPSPPITTTTTPPPTTTPSSPITTTPSPPTTTMTTPSPTTTPSSPITTTTTPSSTTTPSPPPTTMTTPSPTTTPSPPTTTMTTLPPTTTSSPLTTTPLPPSITPPTFSPFSTTTPTTPCVPLCNWTGWLDSGKPNFHKPGGDTELIGDVCGPGWAANISCRATMYPDVPIGQLGQTVVCDVSVGLICKNEDQKPGGVIPMAFCLNYEINVQCCECVTQPTTMTTTTTENPTPPTTTPITTTTTVTPTPTPTGTQTPTTTPITTTTTVTPTPTPTGTQTPTTTPITTTTTVTPTPTPTGTQTPTTTPITTTTTVTPTPTPTGTQTPTTTPITTTTTVTPTPTPTGTQTPTTTPITTTTTVTPTPTPTGTQTPTTTPITTTTTVTPTPTPTGTQTPTTTPITTTTTVTPTPTPTGTQTPTTTPITTTTTVTPTPTPTGTQTPTTTPITTTTTVTPTPTPTGTQTPTTTPITTTTTVTPTPTPTGTQTPTTTPITTTTTVTPTPTPTGTQTPTTTPITTTTTVTPTPTPTGTQTPTTTPITTTTTVTPTPTPTGTQTPTTTPITTTTTVTPTPTPTGTQTPTTTPITTTTTVTPTPTPTGTQTPTTTPITTTTTVTPTPTPTGTQTPTTTPITTTTTVTPTPTPTGTQTPTTTPITTTTTVTPTPTPTGTQTPTTTPITTTTTVTPTPTPTGTQTPTTTPITTTTTVTPTPTPTGTQTPTTTPITTTTTVTPTPTPTGTQTPTTTPITTTTTVTPTPTPTGTQTPTTTPITTTTTVTPTPTPTGTQTPTTTPITTTTTVTPTPTPTGTQTPTTTPITTTTTVTPTPTPTGTQTPTTTPITTTTTVTPTPTPTGTQTPTTTPITTTTTVTPTPTPTGTQTPTTTPITTTTTVTPTPTPTGTQTPTTTPITTTTTVTPTPTPTGTQTPTTTPITTTTTVTPTPTPTGTQTPTTTPITTTTTVTPTPTPTGTQTPTTTPITTTTTVTPTPTPTGTQTPTTTPITTTTTVTPTPTPTGTQTPTTTPITTTTTVTPTPTPTGTQTPTTTPITTTTTVTPTPTPTGTQTPTTTPITTTTTVTPTPTPTGTQTPTTTPITTTTTVTPTPTPTGTQTPTTTPITTTTTVTPTPTPTGTQTPTTTPITTTTTVTPTPTPTGTQTPTTTPITTTTTVTPTPTPTGTQTPTTTPITTTTTVTPTPTPTGTQTPTTTPITTTTTVTPTPTPTGTQTPTTTPITTTTTVTPTPTPTGTQTPTTTPITTTTTVTPTPTPTGTQTPTTTPITTTTTVTPTPTPTGTQTPTTTPITTTTTVTPTPTPTGTQTPTTTPITTTTTVTPTPTPTGTQTPTTTPITTTTTVTPTPTPTGTQTPTTTPITTTTTVTPTPTPTGTQTPTTTPITTTTTVTPTPTPTGTQTPTTTPITTTTTVTPTPTPTGTQTPTTTPITTTTTVTPTPTPTGTQTPTTTPITTTTTVTPTPTPTGTQTPTTTPITTTTTVTPTPTPTGTQTPTTTPITTTTTVTPTPTPTGTQTPTTTPITTTTTVTPTPTPTGTQTPTTTPITTTTTVTPTPTPTGTQTPTTTPITTTTTVTPTPTPTGTQTPTTTPITTTTTVTPTPTPTGTQTPTTTPITTTTTVTPTPTPTGTQTPTTTPITTTTTVTPTPTPTGTQTPTTTPITTTTTVTPTPTPTGTQTPTTTPITTTTTVTPTPTPTGTQTPTTTPITTTTTVTPTPTPTGTQTPTTTPITTTTTVTPTPTPTGTQTPTTTPITTTTTVTPTPTPTGTQTPTTTPITTTTTVTPTPTPTGTQTPTTTPITTTTTVTPTPTPTGTQTPTTTPITTTTTVTPTPTPTGTQTPTTTPITTTTTVTPTPTPTGTQTPTTTPITTTTTVTPTPTPTGTQTPTTTPITTTTTVTPTPTPTGTQTPTTTPITTTTTVTPTPTPTGTQTPTTTPITTTTTVTPTPTPTGTQTPTTTPITTTTTVTPTPTPTGTQTPTTTPITTTTTVTPTPTPTGTQTPTTTPITTTTTVTPTPTPTGTQTPTTTPITTTTTVTPTPTPTGTQTPTTTPITTTTTVTPTPTPTGTQTPTTTPITTTTTVTPTPTPTGTQTPTTTPITTTTTVTPTPTPTGTQTPTTTPITTTTTVTPTPTPTGTQTPTTTPITTTTTVTPTPTPTGTQTPTTTPITTTTTVTPTPTPTGTQTPTTTPITTTTTVTPTPTPTGTQTPTTTPITTTTTVTPTPTPTGTQTPTTTPITTTTTVTPTPTPTGTQTPTTTPITTTTTVTPTPTPTGTQTPTTTPITTTTTVTPTPTPTGTQTPTTTPITTTTTVTPTPTPTGTQTPTTTPITTTTTVTPTPTPTGTQTPTTTPITTTTTVTPTPTPTGTQTPTTTPITTTTTVTPTPTPTGTQTPTTTPITTTTTVTPTPTPTGTQTPTTTPITTTTTVTPTPTPTGTQTPTTTPITTTTTVTPTPTPTGTQTPTTTPITTTTTVTPTPTPTGTQTPTTTPITTTTTVTPTPTPTGTQTPTTTPITTTTTVTPTPTPTGTQTGPPTHTSTAPIAELTTSNPPPESSTPQTSRSTSSPLTESTTLLSTLPPAIEMTSTAPPSTPTAPTTTSGGHTLSPPPSTTTSPPGTPTRGTTTGSSSAPTPSTVQTTTTSAWTPTPTPLSTPSIIRTTGLRPYPSSVLICCVLNDTYYAPGEEVYNGTYGDTCYFVNCSLSCTLEFYNWSCPSTPSPTPTPSKSTPTPSKPSSTPSKPTPGTKPPECPDFDPPRQENETWWLCDCFMATCKYNNTVEIVKVECEPPPMPTCSNGLQPVRVEDPDGCCWHWECDCYCTGWGDPHYVTFDGLYYSYQGNCTYVLVEEISPSVDNFGVY**IDNYHCDPNDKVSCPRTLIVRHETQEVLIK**TVHMMPMQVQVQVNR**QAVALPYKKYGLEVYQSGINY**VVDIPELGVLVSYNGLSFSVRLPYHRFGNNTKGQCGTCTNTTSDDCILPSGEIVSNCEAAADQWLVNDPSKPHCPHSSSTTKRPAVTVPGGGKTTPHKDCTPSPLCQLIKDSLFAQCHALVPPQHYYDACVFDSCFMPGSSLECASLQAYAALCAQQNICLDWRNHTHGACLVECPSHREYQACGPAEEPTCKSSSSQQNNTVLVEGCFCPEGTMNYAPGFDVCVKTCGCVGPDNVPR**EFGEHFEFDCK**NCVCLEGGSGIICQPKRCSQKPVTHCVEDGTYLATEVNPADTCCNITVCKCNTSLCKEKPSVCPLGFEVKSKMVPGRCCPFYWCESK**GVCVHGNAEYQPGSPVYSSK**CQDCVCTDKVDNNTLLNVIACTHVPCNTSCSPGFELMEAPGECCKKCEQTHCIIKRPDNQHVILKPGDFKSDPKNNCTFFSCVKIHNQLISSVSNITCPNFDASICIPGSITFMPNGCCKTCTPRNETRVPCSTVPVTTEVSYAGCTKTVLMNHCSGSCGTFVMYSAKAQALDHSCSCCKEEKTSQREVVLSCPNGGSLTHTYTHIESCQCQDTVCGLPTGTSRRARRSPRHLGSG | 101  202  303  404  505  606  707  808  909  1010  1111  1212  1313  1414  1515  1616  1717  1818  1919  2020  2121  2222  2323  2424  2525  2626  2727  2828  2929  3030  3131  3232  3333  3434  3535  3636  3737  3838  3939  4040  4141  4242  4343  4444  4545  4646  4747  4848  4949  5050  5151  5179 |
| --- | --- |
| **Figure S4.** Muc2 (Sequence ID: Q02817.2) peptide mass fingerprint matches to the SwissProt database from LC-MS/MS analysis of ND-mucin preparation with matched peptides highlighted in color and bold. Due to the incomplete porcine genome, predictions of porcine Muc2 matched to the human MUC2 accession. | |

**Figure S5**

| **A** | 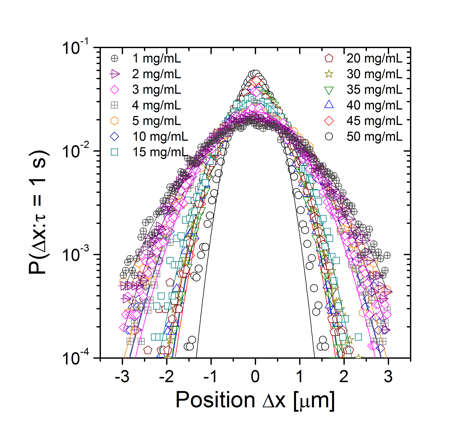 | **B** | 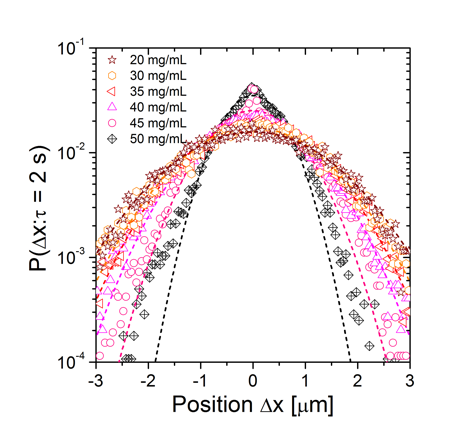 |
| --- | --- | --- | --- |
| **C** | 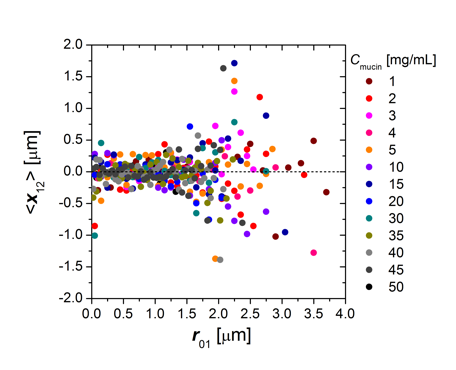 | **D** | 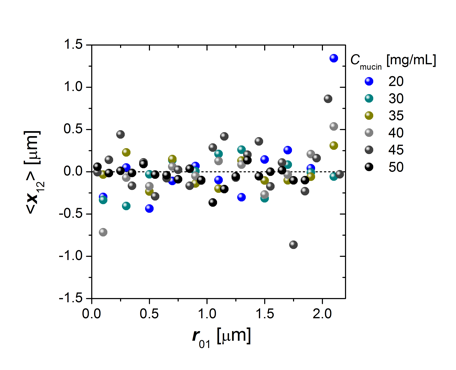 |
| **Figure S5.** Microrheological heterogeneity characterization of ND-mucin solutions. A.) Probability distributions of 0.5 μm colloidal particle movement by Δ*x*-distances with a lag time of 1 s for mucin concentrations from 1 ≤ c ≤ 50 mg/mL. The solid lines represent Gaussian distributions fitted to the data. B.) Probability distributions of 0.5 μm colloidal particle movement by Δ*x*-distances with a lag time of 2 s for mucin concentrations from 20 ≤ c ≤ 50 mg/mL. The solid lines represent Gaussian distributions fitted to the data. C.) Degree of correlation between successive particle displacements for a lag time of 1 s for mucin concentrations from 1 ≤ c ≤ 50 mg/mL. Dashed line at the zero position corresponds to no correlation between successive particle displacements. C.) Degree of correlation between successive particle displacements for lag time of 2 s and mucin concentrations from 20 mg/mL to 50 mg/mL. Dashed line at the zero position corresponds to no correlation between successive particle displacements | | | |

**Figure S6**

|  | 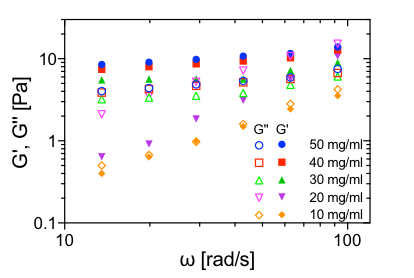 |  |  |
| --- | --- | --- | --- |
| **Figure S6.** Comparison of the storage, G’ (ω), and loss moduli, G’’ (ω) of 10 to 50 mg/mL ND-mucin. For concentrations below 10 mg/mL, the steady shear profiles were within the standard error from that of pure buffer. | | | |

**Figure S7**

|  |
| --- |
| **Figure S7.** Rheological properties of porcine small intestinal mucus measured shortly after sacrifice. (A) Shear viscosity. (B) Yield stress. Storage (G’; black circles) and loss (G’’; white circles) modulus as a function of (C) oscillatory frequency and (D) stress. |

**Figure S8**

| **A** | 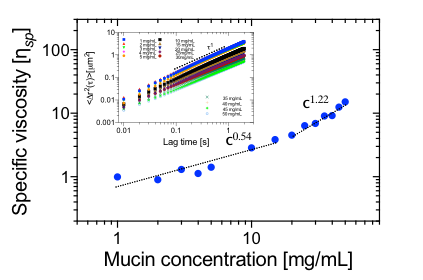 | **B** | 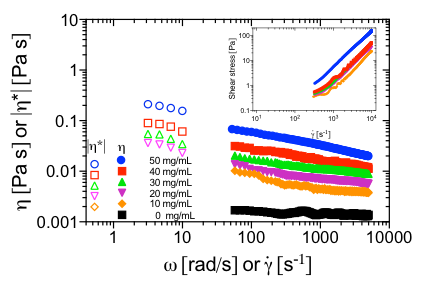 |
| --- | --- | --- | --- |
| **C** | 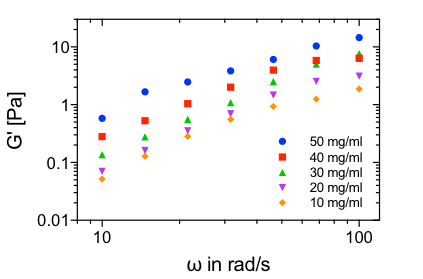 | **D** | 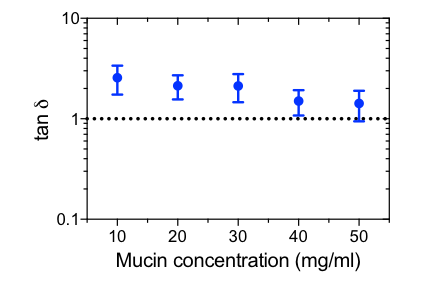 |
| **Figure S8.** Rheological characterization of GH-mucin solutions. A.) Specific viscosity ($\eta_{sp}$) scaling of 1 ≤ c ≤ 50 mg/mL GH-mucin. Insert: The ensemble averaged MSD curves of 0.5 μm colloidal particles in 1 ≤ c ≤ 50 mg/mL GH-mucin. A single regime was observed in GH-mucin based on the power law of the MSD curves showing the log-log scaling of τ^1^. B.) Steady shear viscosity, η, and complex viscosity, \|η*\|, of 10 ≤ c ≤ 50 mg/mL GH-mucin versus shear rate, $\dot{\gamma}$, and angular frequency, ω, measured using parallel-plate geometry at a gap of 40 μm. Insert shows the plot of shear stress, σ, versus shear rate, $\dot{\gamma}$, of the same data. For concentrations below 10 mg/mL, the steady shear profiles were within the standard error from that of pure buffer. C.) Comparison of the storage moduli, G’ (ω) of 10 ≤ c ≤ 50 mg/mL GH-mucin. For concentrations below 10 mg/mL, the steady shear profiles were within standard error from that of pure buffer. D.) The loss tangent (tan δ), the ratio of loss modulus (G’’) to storage modulus (G’), of 10 ≤ c ≤ 50 mg/mL GH-mucin. | | | |

**Figure S9**

| **A** |  | **B** | 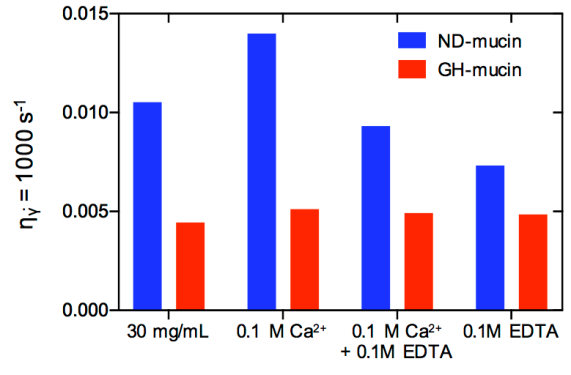 |
| --- | --- | --- | --- |
| **C** | 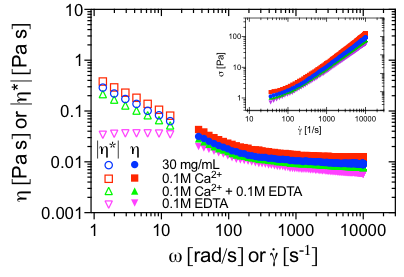 | **D** | 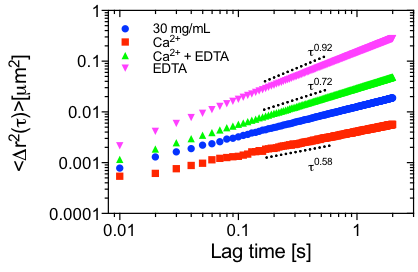 |
| **E** | 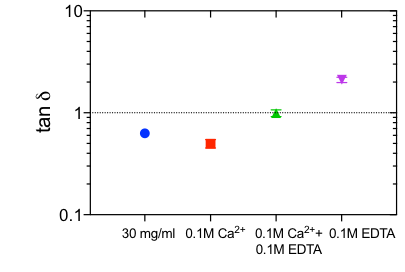 |  |  |
| **Figure S9.** A.) Steady shear viscosity, η, of 30 mg/mL ND-mucin (closed symbols) and GH-mucin (open symbols) treated with 0.1 M Ca^2+^, 0.1 M Ca^2+^/0.1 M EDTA and 0.1 M EDTA graphed versus shear rate, $\dot{\gamma}$, measured using parallel-plate geometry at a gap of 40 μm. B.) Steady shear viscosity, η, at a shear rate of 1000 s^-1^ of 30 mg/mL ND- and GH-mucin treated with 0.1 M Ca^2+^, 0.1 M Ca^2+^/0.1 M EDTA and 0.1 M EDTA. C.) Steady shear viscosity, η, and oscillatory viscosity, \|η*\|, of 30 mg/mL ND-mucin treated with 0.1 M Ca^2+^, 0.1 M Ca^2+^/0.1 M EDTA and 0.1 M EDTA graphed versus shear rate, $\dot{\gamma}$, and angular frequency, ω, measured using parallel-plate geometry at a gap of 40 μm (separate batch cf. shown in Figure 6). D.) The ensemble averaged MSD curves of 0.5 μm colloidal particles in 30 mg/mL ND-mucin treated with 0.1 M Ca^2+^, 0.1 M Ca^2+^/ 0.1M EDTA and 0.1M EDTA, the log-log scaling to be τ^0.62^, τ^0.58^, τ^0.72^ and τ^0.92^, respectively. E.) The loss tangent (tanδ), a ratio of the loss modulus (G’’) to storage modulus (G’) of 30 mg/mL ND-mucin treated with 0.1 M Ca^2+^, 0.1 M Ca^2+^/0.1M EDTA and 0.1 M EDTA. | | | |

**Figure S10**

| **A** | 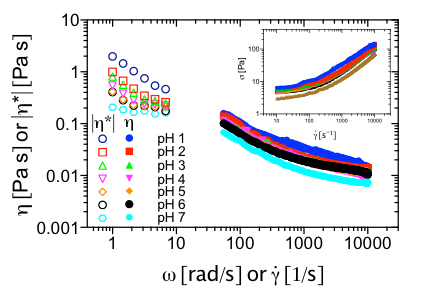 | **B** | 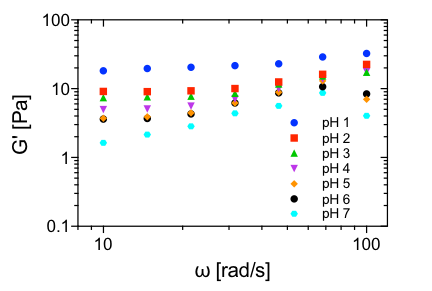 |
| --- | --- | --- | --- |
| **Figure S10.** The effect of pH on rheological properties of ND-mucin solutions. A.) Steady shear viscosity, η, and complex viscosity, \|η*\|, of 30 mg/mL ND-mucin treated with 10 mM 1-7 pH buffer versus shear rate, $\dot{\gamma}$ and angular frequency, ω, measured using parallel-plate geometry at a gap of 40 μm. Insert represents the yield stress, σ, versus shear rate, $\dot{\gamma}$. B.) Comparison of the storage modulus, G’ (ω) of 30 mg/mL ND-mucin treated with 10 mM 1-7 pH buffers. | | | |

**Figure S11**

|  | 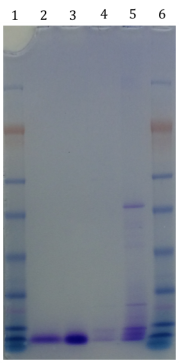 |  |  |
| --- | --- | --- | --- |
| **Figure S11.** Complete figure of SDS-PAGE of ND-mucin and GH-mucin preparations of porcine intestinal mucin after two step CsCl isopycnic density gradient centrifugation. Molecular marker (lanes: 1 and 6), reduced ND-mucin (lane: 5), non-reduced ND-mucin (lane: 4), reduced GH-mucin (lane: 2) and non-reduced GH-mucin (lane: 3). | | | |

**References**

Ambort, D., van der Post, S., Johansson, Malin E V., MacKenzie, J., Thomsson, E., Krengel, U., & Hansson, Gunnar C. (2011). Function of the CysD domain of the gel-forming MUC2 mucin. *Biochemical Journal, 436*(Pt 1), 61-70. doi:10.1042/BJ20102066

Davies, J. R., Wickstrom, C., & Thornton, D. J. (2012). Gel-Forming and Cell-Associated Mucins: Preparation for Structural and Functional Studies. In M. A. McGuckin & D. J. Thornton (Eds.), *Mucins - Methods and Procedures*: Springer Science+Business Media.

Di Cola, E., Yakubov, G. E., & Waigh, T. A. (2008). Double Globular Structure of Porcine Stomach Mucin: A Small-Angle X-ray Scattering Study. *Biomacromolecules, 9*(11), 3216-3222.

Georgiades, P., di Cola, E., Heenan, R. K., Pudney, P. D. A., Thornton, D. J., & Waigh, T. A. (2014). A Combined Small-Angle X-ray and Neutron Scattering Study of the Structure of Purified Soluble Gastrointestinal Mucins. *Biopolymers, 101*(12), 1154-1164. doi:10.1002/bip.22523

Georgiades, P., Pudney, P. D. A., Thornton, D. J., & Waigh, T. A. (2014). Particle Tracking Microrheology of Purified Gastrointestinal Mucins. *Biopolymers, 101*(4), 366-377. doi:10.1002/bip.22372

Kegel, W. K., & van Blaaderen, A. (2000). Direct observation of dynamical heterogeneities in colloidal hard-sphere suspensions. *Science, 287*(5451), 290-293. doi:10.1126/science.287.5451.290

Matsuo, K., Ota, H., Akamatsu, T., Sugiyama, A., & Katsuyama, T. (1997). Histochemistry of the surface mucous gel layer of the human colon. *Gut, 40*(6), 782-789.

Ridley, C., Kouvatsos, N., Raynal, B. D., Howard, M., Collins, R. F., Desseyn, J. L., . . . Thornton, D. J. (2014). Assembly of the Respiratory Mucin MUC5B: A new model for a gel-forming mucin. *Journal of Biological Chemistry, 289*(23), 16409-16420. doi:10.1074/jbc.M114.566679

Rubinstein, M., Colby, R. H., Dobrynin, A. V., & Joanny, J. F. (1996). Elastic modulus and equilibrium swelling of polyelectrolyte gels. *Macromolecules, 29*(1), 398-406. doi:10.1021/ma9511917

Schutte, A., Ermund, A., Becker-Pauly, C., Johansson, M. E., Rodriguez-Pineiro, A. M., Backhed, F., . . . Hansson, G. C. (2014). Microbial-induced meprin beta cleavage in MUC2 mucin and a functional CFTR channel are required to release anchored small intestinal mucus. *Proc Natl Acad Sci U S A, 111*(34), 12396-12401. doi:10.1073/pnas.1407597111

Verdugo, P., Aitken, M., Langley, L., & Villalon, M. J. (1987). Molecular mechanism of product storage and release in mucin secretion. II. The role of extracellular Ca++. *Biorheology, 24*(6), 625-633.
